# The structural basis of CstF-77 modulation of cleavage and polyadenylation through stimulation of CstF-64 activity

**DOI:** 10.1093/nar/gky862

**Published:** 2018-09-26

**Authors:** Petar N Grozdanov, Elahe Masoumzadeh, Michael P Latham, Clinton C MacDonald

**Affiliations:** 1 Department of Cell Biology & Biochemistry, School of Medicine, Texas Tech University Health Sciences Center, Lubbock, TX 79430-6540, USA; 2 Department of Chemistry and Biochemistry, Texas Tech University, Lubbock, TX 79409-1061, USA

## Abstract

Cleavage and polyadenylation (C/P) of mRNA is an important cellular process that promotes increased diversity of mRNA isoforms and could change their stability in different cell types. The cleavage stimulation factor (CstF) complex, part of the C/P machinery, binds to U- and GU-rich sequences located downstream from the cleavage site through its RNA-binding subunit, CstF-64. Less is known about the function of the other two subunits of CstF, CstF-77 and CstF-50. Here, we show that the carboxy-terminus of CstF-77 plays a previously unrecognized role in enhancing C/P by altering how the RNA recognition motif (RRM) of CstF-64 binds RNA. In support of this finding, we also show that CstF-64 relies on CstF-77 to be transported to the nucleus; excess CstF-64 localizes to the cytoplasm, possibly via interaction with cytoplasmic RNAs. Reverse genetics and nuclear magnetic resonance studies of recombinant CstF-64 (RRM-Hinge) and CstF-77 (monkeytail-carboxy-terminal domain) indicate that the last 30 amino acids of CstF-77 increases the stability of the RRM, thus altering the affinity of the complex for RNA. These results provide new insights into the mechanism by which CstF regulates the location of the RNA cleavage site during C/P.

## INTRODUCTION

Diversity in the transcriptome is achieved through alternative transcription start sites, alternative exon splicing and alternative 3′ end processing. As such, 3′ end processing is an important regulatory mechanism in normal development or disease states (1–4). 3′ end processing of most mRNAs involves cleavage of nascent mRNA followed by addition of a homopolymeric tail of 150–250 adenosine residues to the upstream RNA product, a process known as cleavage and polyadenylation (C/P). Alternative C/P can modulate the length of the 3′ untranslated region (UTR), truncate open reading frames or encode new protein domains into an mRNA transcript (5,6). For example, mRNAs with shorter 3′ UTRs are associated with proliferative cells and cells that are prone to malignant transformation (7,8). In metazoans, C/P is regulated mainly through a *cis*-RNA sequence known as the polyadenylation signal. The polyadenylation signal is upstream of the cleavage site, and consists of six nucleotides, the most common being AAUAAA (5,9,10). The polyadenylation signal is recognized by the Cleavage and Polyadenylation Specificity Factor (CPSF) complex (including two proteins, CPSF-30 and WDR33 that recognize the polyadenylation signal (11,12)) and the endonuclease, CPSF-73 (13).

A second protein complex, the cleavage stimulation factor, CstF, is involved in regulation of 3′ end processing by recognizing the correct site for C/P. CstF consists of three proteins, CstF-50 (gene symbol, *CSTF1*), CstF-64 *(CSTF2)*, and CstF-77 *(CSTF3)*. The downstream sequence element (DSE) is located about 10–24 nt 3′ of the C/P site and is composed of U- or GU-rich sequences. CstF recognizes the DSE through CstF-64 (14), which contains an RNA recognition motif (RRM) at its amino-terminus (15).

Nuclear magnetic resonance (NMR) and X-ray crystallography studies identified RNA-binding surfaces of the human RRM (16,17) and the yeast homolog of CstF-64, Rna15p (18,19). In both cases, U-rich ribonucleotide sequences bind to a central β-sheet in the RRM, and modulation of secondary structures surrounding the central β-sheet allows CstF-64 to bind to additional G and U residues, while still being able to discriminate against As and Cs. The Hinge domain is adjacent to the RRM, and is involved in interactions with CstF-77 (20–22) and symplekin (23) in a mutually exclusive fashion (24). Other domains of CstF-64 mediate interactions with other nuclear functions including splicing and transcriptional termination (23,25–27). In addition to its functions in mRNA C/P, CstF-64 is involved in histone mRNA 3′ end formation, whereby it governs the G_1_ to S transition in cell cycle (28,29). Tissue-specific variants of CstF-64 are expressed in brain and testis, where they subsume or complement its normal functions (30–36).

The CstF complex has a hexameric architecture, consisting of a dimer of two trimeric CstF subunits (37–40), which seems to play an important role in the recognition of the DSE (41). One role of CstF-77 in the complex is to act as a scaffold linking CstF-64 and CstF-50 to the CPSF (42). CstF-77 is comprised of twelve half-a-tetratricopeptide repeat (HAT) domains and a proline-rich protein-protein interaction domain called the ‘monkeytail’ (21). The monkeytail (MT) has been shown to interact with the Hinge domain of CstF-64 (21,37). In addition, a region adjacent to the MT is involved in interactions with CstF-50, suggesting a range of flexibility and conformational positions between the CstF-64, CstF-50 and CstF-77 proteins.

CstF-77 is the protein in the CstF complex possessing a monopartite nuclear localization signal (NLS, 22). This NLS is necessary for transport of the entire complex into the nucleus (20). In the cytoplasm, CstF-77 is found as part of the translational masking complex, with the result that impairment of the CstF-77 synthesis leads to acceleration of the G_2_/M transition (43).

In the current study, we examined mechanisms by which CstF-64 and CstF-77 cooperate to regulate cleavage and polyadenylation. Exogenous expression of CstF-64 results in increased C/P of a reporter gene in correlation with the amount of CstF-64 protein. Co-expression of both CstF-77 and CstF-50 with CstF-64 further increased C/P of the reporter gene. As previously shown (20), we confirm that one role of CstF-77 is to transport the CstF complex into the nucleus. However, we discovered a previously uncharacterized interaction between CstF-64 and CstF-77 that specifically enhances C/P of the reporter gene by altering RNA binding by CstF-64. Using NMR spectroscopy, we demonstrate that the last 30 amino acids of CstF-77, which are conserved from *Drosophila melanogaster* to humans but absent in *Saccharomyces cerevisiae*, alter the binding of the complex to RNA *in vitro* and increase C/P *in vivo*. These results establish the regulatory function of the CstF complex and provide a mechanism by which CstF-77 can influence cleavage and polyadenylation site selection in all metazoans.

## MATERIALS AND METHODS

### Cell line, cell culture, transfection and treatment of the cells

In all of our experiments we used HeLa cells purchased from the American Type Culture Collection. The first plating of the cells in our lab was designated as P1. We used the cells in our experiments up to P15 without noticing any changes in the outcomes of our assays. HeLa cells were grown in Dulbecco’s modified Eagle’s medium supplemented with 10% fetal bovine serum,10 I.U./ml penicillin and 10 μg/ml streptomycin. Cells were maintained in an incubator supplemented with 5% CO_2_ at 37°C.

Transfection experiments were performed in 24-well plate and 10 cm dishes. A single well of a 24-well plate was seeded with 40 000 cells and grown overnight. The next day cells were transfected with the appropriate combination of plasmids premixed with the Lipofectamine LTX reagent (ThermoFisher Scientific) exactly as outlined in the manufacture's manual. This procedure was repeated as many times as needed for our experiments.

Knock down experiments were performed using Lipofectamine RNAiMAX reagent (ThermoFisher Scientific) and siRNAs (OriGene) as specified by the manufacturer.

Transfected HeLa cells were treated with 20 nM Leptomycin B (Cell Signaling Technology) for either 3 or 16 h. After completion of the treatment the cells were either fixed with 4% paraformaldehyde or processed for SLAP (see below).

### Plasmids used in the study

All plasmids were verified by Sanger sequencing before use. pGL3 plasmid (Promega) contains *Photinus* luciferase and was used as a normalization control as previously reported (20,44). *Renilla-*luciferase construct (SL-Luc) containing the modified cleavage and polyadenylation site with the addition of two MS2 stem-loop sequences downstream of the C/P site was used also as described. SL-Luc construct is a derivative of pRL-SV40 plasmid (Promega).

All CstF-64 constructs were based on a previously published construct, MCP-CstF-64 (45). All derivatives and mutants of MCP-CstF-64 constructs were created through site directed mutagenesis, either using the QuikChange Lightning Multi Site-Directed Mutagenesis Kit (Agilent) or complementary pair of primers and Q5 polymerase (New England Biolabs), followed by a digestion with the DpnI enzyme. MCP-CstF-64-ΔHinge was identical to MS2-CstF-64_ΔHinge_ from Hockert *et al.* (20). MCP-CstF-64(R159P) was created using site directed mutagenesis based on the work of Hockert *et al.* (20). MCP-CstF-64(H4) was based on the Hydro H4 mutant of Ruepp *et al.* (46), and consists of mutations to L168G, L172G, L173G, M179G, I181G. Replacement of RRM of CstF-64 in MCP-CstF-64 construct with the SUMO-domain (47) was performed by using a megaprimer approach.

Open reading frame of human CstF-77, transcript variant 1(accession number: NM_001326.2) was cloned in pcDNA 3.1/Myc-His in frame with Myc-His tag using restriction enzymes. However, our preliminary experiments demonstrated that the in-frame Myc tag was not detectable by a western blot. Therefore, we introduced two additional Myc tags using megaprimer approach to create CstF-77-Myc construct. Deletion of the MT (amino acids 607–664) and part of the carboxy-terminal domain (CTD) (amino acids 688–717) of CstF-77 was performed using site directed mutagenesis and CstF-77-Myc construct as a template.

Human CstF-50 open reading frame was cloned in pcDNA 3.1/Myc-His containing three HA tags at the amino-terminal end of the protein and a stop codon before the Myc-tag.

The RRM (amino acids 1–107) of CstF-64 was cloned in a modified vector based on pET22 as a fusion with a six-histidine tag followed by a tobacco etch virus (TEV) protease site at the amino terminal end of the RRM. The sequences for the RRM-Hinge of CstF-64 (amino acids 1–195) and MT-CTD of CstF-77 (amino acids 607–717) were cloned in modified version of pMal-C with a bicistronic design. The RRM-Hinge, in the bicistronic expression vector, is a fusion with a six-histidine tag on the amino terminal side of the protein followed by a TEV site. The MT-CTD of CstF-77 is a fusion with a maltose binding protein separated by a TEV site. The deletion of the last 30 amino acids of CstF-77 in the construct was performed using a site directed mutagenesis.

### Stem-loop assay for polyadenylation, SLAP

SLAP was performed as previously described (20,44). As a substrate for the *Renilla* and *Photinus* luciferases we used Dual-Luciferase Reporter Assay Kit (Promega). The regents were prepared as recommended by the supplier. Transfected cells were washed once in PBS (phosphate-buffered saline). Transfected cells from a single 24-well were lysed in 100 μl of 1× Passive Lysis Buffer for 15 min with a gentle agitation. Between 2 and 5 μl of the lysates were used to measure the luciferases activity from each sample in a DLReady validated luminometer (TD-20/20, Turner designs) as recommended by the Dual-Luciferase Reporter Assay Kit manual. The luminometer was programed to perform a 2-s premeasurement delay, followed by a 10-s measurement period for each assay. The measurement of the each of the luciferase assays were recorded. SLAP was performed in a triplicate for any given combination of plasmids. Average normalized values were plotted using Microsoft Excel software. The standard error was calculated by dividing the standard deviation by the square root of the total number of measurements of the normalized SLAP values. Statistical significance was determined using a *t*-test with a two-tailed distribution and an unequal variance of the two samples.

### Protein isolation and immunoblots

Immunoblots were performed on the protein samples collected for the luciferase measurements. Between 5 and 10 μl of the samples were loaded on 8 to 10% homemade sodium dodecyl sulphate-polyacrylamide gel electrophoresis (SDS-PAGE). The resolved proteins were transferred to a PVDF membrane through semi-dry transfer. After the completion of the transfer PVDF membranes were stained with Ponceau S to ensure even and complete transfer of the protein samples. PVDF membranes were blocked with 5% non-fat milk in TBST buffer (20 mM Tris–HCl pH 7.6, 150 mM NaCl, 0.05% Tween-20) for 1 h at room temperature with gentle agitation. Different antibodies were applied either in a dilution as per the recommendation of the supplier or as previously reported (48) in 2% non-fat milk/ TBST buffer overnight at 4°C. The next day the membranes were washed three times in TBST and incubated with the appropriate secondary horseradish peroxidase conjugated antibody. After a second round of three washed the protein of interest were visualized using the SuperSignal West Pico Chemiluminescence kit (ThermoFisher Scientific) and images were taken on ImageQuant LAS 4000 imager. Whenever possible, the membranes were split and different parts were incubated with more than one antibody, e.g. a single membrane was split and used to probe for expression of MCP-CstF-64 and β-tubulin across the samples. The rabbit monoclonal anti-Myc antibody (clone 71D10, Cell Signaling Technology) was used in 5% bovine serum albumin instead of 5% non-fat milk.

### Immunofluorescence and quantification of nuclear/cytoplasm ratios

Immunofluorescence experiments were performed as described previously (49). HeLa cells were plated on 12 mm borosilicate glass coverslips in 24-well plate and transfected as described above. Transfected HeLa cells were taken out of the incubator, growth media was removed and the cells were immediately fixed in 4% paraformaldehyde in PBS for 20 min at room temperature. Cells were washed three times with PBS and permeabilized for 10 min in 1% Triton X-100, PBS. Unspecific binding sites were blocked with 1% non-fat milk in PBS for 15 min at room temperature. Primary antibodies against FLAG and Myc tags (detecting MCP-CstF-64 and CstF-77 constructs, and derivatives, respectively) were diluted 1:200 and applied on the cells in 1% non-fat milk/PBS for ∼2 h. Cells were then washed three times in 1% non-fat milk in PBS and incubated with a mixture (1:200 dilution) of donkey anti-mouse Alexa488-conjugated and donkey anti-rabbit Cy3-conjugated secondary antibodies for 40 min at room temperature. Three final washes were performed in 1% non-fat milk/ PBS followed by a single wash in PBS. Cells were mounted using ProLong Diamond Antifade Mountant (ThermoFisher Scientific). Samples were cured overnight at room temperature and sealed permanently with nail polish. Microscopy was performed on an inverted Nikon Ti microscope using a confocal A1 module. Z-stacks were obtained and the final image was assembled as a maximum intensity projection. Final maximum intensity projection images were adjusted for brightness and contrast using ImageJ software.

Quantification of the nuclear localization of CstF-64 and CstF-77 was performed by positioning three measuring squares on the maximum intensity projection image within the nucleus and additional three squares in the cytoplasm of the same cell using NIS Elements software (Nikon Instruments). DAPI staining was used to ensure proper nuclear positioning of the three ‘nuclear’ squares. The intensity of the channels corresponding to Alexa488 and Cy3 was measured for the six squares and the ratio between the nucleus and cytoplasm (nuclear ratio) was calculated. The average nuclear ratio from at least 30 individual cells is displayed per sample. The standard error was calculated by dividing the standard deviation by the square root of the total number of observations of the nuclear ratio. Statistically significance was calculated using a *t*-test with a two-tailed distribution and an unequal variance of the two samples.

### Co-immunoprecipitation

To verify the interactions between the CstF-64 and CstF-77 proteins and their respective mutants we took a co-immunoprecipitation approach. About 2 μg of antibody (9E10 clone, mouse monoclonal, EMD Millipore) against Myc-tag were coupled to Dynabeads Protein G particles (ThermoFisher Scientific). We pooled together the triplicate samples from the SLAP and diluted them with an equal volume of 2 × NP-40 buffer (100 mM Tris–HCl pH 7.4, 300 mM NaCl, 2% NP-40). The diluted lysate was applied on the magnetic beads and incubated in a thermomixer at 4°C for 1 h. The magnetic beads were washed five times in 1 × NP-40 buffer and once in PBS. The protein samples were released from the beads by incubation in SDS-PAGE loading buffer for 5 min at 95°C. A third to about a half of the immunoprecipitates were loaded on SDS-PAGE and were processed for immunoblots.

### Crosslinking and immunoprecipitation experiments, CLIP

To determine the interaction between the MCP-CstF-64 mutants and RNA, we performed cross-linking and immunoprecipitation followed by SDS-PAGE. Briefly, MCP-CstF-64 wild-type and RRM mutants were transiently transfected in HeLa cells in 10 cm dishes using scaled-up lipofectamine protocol. Forty-eight hours post transfection cells were UV-crosslinked, lysed in RIPA buffer and the rest of the CLIP procedure was performed exactly as previously described (50) using anti-FLAG-specific (M2 clone, SigmaAldrich) monoclonal antibody. CLIP for CstF-77-Myc and deletion mutants was performed in the same fashion using anti-Myc-specific (9E10, EMD Millipore) antibody. CLIP protocol was stopped after the development of the radioactive gel.

### Bacterial protein expression and purification

The constructs were transformed in Rosetta™ 2 (DE3) pLysS cell (Novagen). Multiple colonies were used to inoculate LB medium supplemented with 100 μg/ml ampicillin and 34 μg/ml chloramphenicol. The cells were grown at 37°C to an optical density of 1. Portion of the cells were used to start an overnight culture in M9 minimal media using ^15^NH_4_Cl and unlabeled d-glucose as the sole nitrogen and carbon sources, respectively. One-liter cultures were induced with 0.5 mM isopropyl-β-d-thiogalactopyranoside (IPTG) at an optical density of 0.8 at 37°C for 4 h. Bacterial cells were suspended in either 1 M NaCl, 25 mM Tris–HCl pH 7.5, 1 mM EDTA, 0.05% sodium azide for the constructs containing the maltose binding protein (see above) or 1 M NaCl, 20 mM HEPES pH 7.5, 5 mM Imidazole, 0.05% sodium azide for the RRM. Cell suspensions were lysed in a microfluidizer processor M-110EH (Microfluidics). Lysates were clarified by centrifugation at 16 000 × *g* for 20 min at 4°C and loaded on home-packed columns either with an Amylose High Flow resin (New England Biolabs) or TALON Superflow Metal Affinity resin (TaKaRa) pre-equilibrated with the corresponding lysis buffers using peristaltic pump. Bound protein was washed with about 10 column volumes in the corresponding lysis buffer followed by a wash with about five column volumes in buffer containing 0.3 M NaCl, 20 mM HEPES pH 7.5, 5 mM imidazole, 0.05% sodium azide. MBP constructs were eluted in 0.3 M NaCl, 20 mM HEPES pH 7.5, 5 mM imidazole, 0.05% sodium azide supplemented with 10 mM maltose. The His-tag bearing RRM was eluted in the above buffer containing 200 mM imidazole. Prior to NMR spectroscopy, the His and MBP-tags were removed with the addition of TEV protease. The proteins were further purified by passing over a HisTrap HP column (GE Biosciences) and were buffer exchanged and concentrated to a desirable concentration for NMR spectroscopy using a 5 kDa molecular weight cutoff centrifugal concentrator (Millipore).

### NMR spectroscopy and binding affinity calculations

NMR experiments were performed on an Agilent 600 MHz (14.1 T) DD2 NMR spectrometer equipped with a room temperature HCN *z*-axis gradient probe. Two-dimensional gradient-selected, sensitivity-enhanced ^1^H-^15^N heteronuclear single quantum correlation (HSQC) (51,52) spectra were collected in 10 mM phosphate buffer pH 6.0 and 2mM TCEP at 30°C. NMR data were processed with NMRPipe (53) and analyzed with CCPN Analysis (54). Amide peak assignments for the RRM were taken from the previous assignments of Varani and co-workers ((16,17), BRMB id: 5774). RNA titration experiments were performed by adding unlabeled RNA derived from the SV40 late transcription unit (14) (5′-AUUUUAUGUUUCAGGU-3′ purchased from SigmaAldrich) to the ^15^N-labeled proteins until complete saturation, as indicated by the end of changes in the peak positions of the ^1^H-^15^N HSQC spectra. Affinity values of the proteins for the RNA were calculated by fitting the RRM peaks in the fast exchange regime to

\begin{eqnarray*}&&\Delta \delta _i^{NH} =\nonumber\\ &&\Delta \delta _{{\rm max}}^{NH}\frac{{\left( {{K_{\rm D}} + {{\left[ P \right]}_{\rm T}} + {{\left[ {{\rm RNA}} \right]}_{\rm T}}} \right) - \ \sqrt {{{\left( {{K_{\rm D}} + {{\left[ P \right]}_{\rm T}} + {{\left[ {{\rm RNA}} \right]}_{\rm T}}} \right)}^2} - 4{{\left[ P \right]}_{\rm T}}{{\left[ {{\rm RNA}} \right]}_{\rm T}}} }}{{2\ {{\left[ P \right]}_{\rm T}}}}\end{eqnarray*}

where $\Delta \delta _i^{NH}$ and $\Delta \delta _{{\rm max}}^{NH}$ are the weighted averaged ^1^H/^15^N chemical shift differences of the i-th titration point and RNA free state (e.g. $\Delta \delta _i^{NH} = \ \sqrt {( {{{( {\delta _i^N - \delta _{{\rm free}}^N} )}^2}/25 + {{( {\delta _i^H - \delta _{{\rm free}}^H} )}^2}} )/2}$ and the fully RNA bound state and apo state (e.g. $\Delta \delta _{{\rm max}}^{NH} = \ \sqrt {( {{{( {\delta _{{\rm bound}}^N - \delta _{{\rm free}}^N} )}^2}/25 + {{( {\delta _{{\rm bound}}^H - \delta _{{\rm free}}^H} )}^2}} )/2}$, respectively (55) *K*_D_ is the binding affinity, [P]_T_ is the total concentration of the protein and [RNA]_T_ is the total concentration of SVL RNA (56). The reported *K*_D_ values were obtained from a global fit of the titration data for each protein condition using residue specific $\Delta \delta _{{\rm max}}^{NH}$ values. Errors in the *K*_D_ were derived from the covariance matrix of the fit.

## RESULTS

### Increased SLAP values correlate with increased amounts of MCP-CstF-64

**Figure 1.**
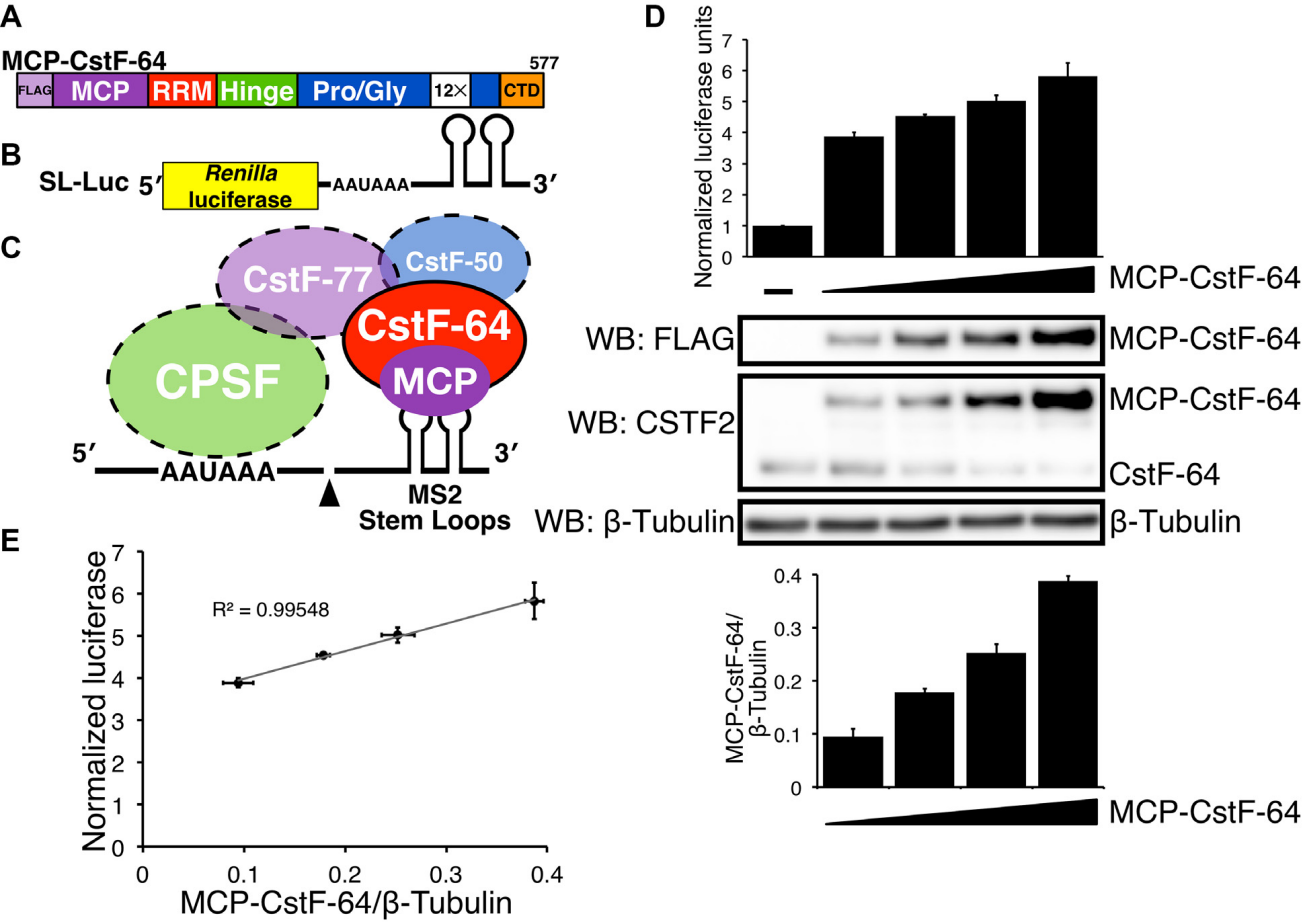
CstF-64 stimulates cleavage and polyadenylation in correlation to the amount of CstF-64 protein produced in SLAP. (**A**) Schematic representation of the coat binding protein from bacteriophage MS2 (MCP) fused to human CstF-64 (MCP-CstF-64). Functional domains in CstF-64 and three FLAG tags are indicated. (**B**) Schematic representation of the reporter construct (SL-Luc) containing the *Renilla* luciferase and two MS2 stem-loop (SL) sequences downstream of the cleavage and polyadenylation site. (**C**) The interaction between MCP-CstF-64 and the SL-Luc reporter construct is mediated through the interaction between MCP and MS2 stem-loops. (**D**) SLAP results showing NLUs in HeLa cells without MCP-CstF-64 (–), and in cells transfected with increasing amounts of MCP-CstF-64. Western blots with the designated antibodies to show the expression of the FLAG-tagged version of MCP-CstF-64 (WB: FLAG) and endogenous CstF-64 (WB: CSTF2). β-tubulin was used as a loading control. A bar graph of the quantified expression of MCP-CstF-64 normalized to β-tubulin (bottom). (**E**) A linear regression between the amount of MCP-CstF-64 protein normalized to β-tubulin (*x*-axis) to the NLU from SLAP (*y*-axis). The correlation value (*R*^2^ = 0.99548) is shown.

Our lab previously developed the Stem-Loop Assay for Polyadenylation (SLAP) to test the role of CstF-64 in C/P without interference from endogenous CstF-64 (20). SLAP relies on a modified version of *Renilla* luciferase (SL-Luc) in which the DSE is replaced by two stem-loop RNA sequences derived from the MS2 bacteriophage (Figure 1B) to stimulate the cleavage of the nascent transcript of the simian vacuolating virus 40 (SV40) late gene (14) followed by polyadenylation of the upstream mRNA (Figure 1C). SLAP relies on a modified CstF-64 that contains an MS2 coat protein (MCP) at its amino-terminal end (MCP-CstF-64, Figure 1A) with three FLAG epitope tags located upstream of the MCP (45). We normalized relative expression of *Renilla* luciferase to *Photinus* (firefly) luciferase (44), which contains an unmodified version of the C/P sequence from the SV40 late transcription unit. Control SLAP samples containing the SL-Luc and firefly constructs but not the MCP-CstF-64 plasmid were included to determine the background level of cleavage and polyadenylation of the SL-Luc reporter. All control samples were normalized to one normalized luciferase unit (NLU).

To confirm that MCP-CstF-64 stimulates C/P, we transfected HeLa cells with increasing amounts of the MCP-CstF-64 plasmid while keeping the SL-Luc construct constant, increasing MCP-CstF-64 to about five times that of the endogenous CstF-64 (Figure 1D). Normalized luciferase expression similarly increased about 5-fold above vector-transfected control cells. A linear regression model showed an almost perfect correlation (*R*^2^ = 0.9955), suggesting that stimulation of C/P depended on MCP-CstF-64 amounts (Figure 1E).

We also noted that increased expression of MCP-CstF-64 led to decreased expression of endogenous CstF-64 (Figure 1D), as was previously observed by us and others (24,30,46). Though untested, we believe that this is due to co-translational regulation of CstF-64 abundance, possibly through interaction with CstF-77 (28). This further confirmed that CstF-64 abundance was critical for C/P and provides a mechanism by which increases or decreases in CstF-64 could regulate alternative C/P (57,58).

### CstF-77 and CstF-50 stimulate C/P through interaction with CstF-64

**Figure 2.**
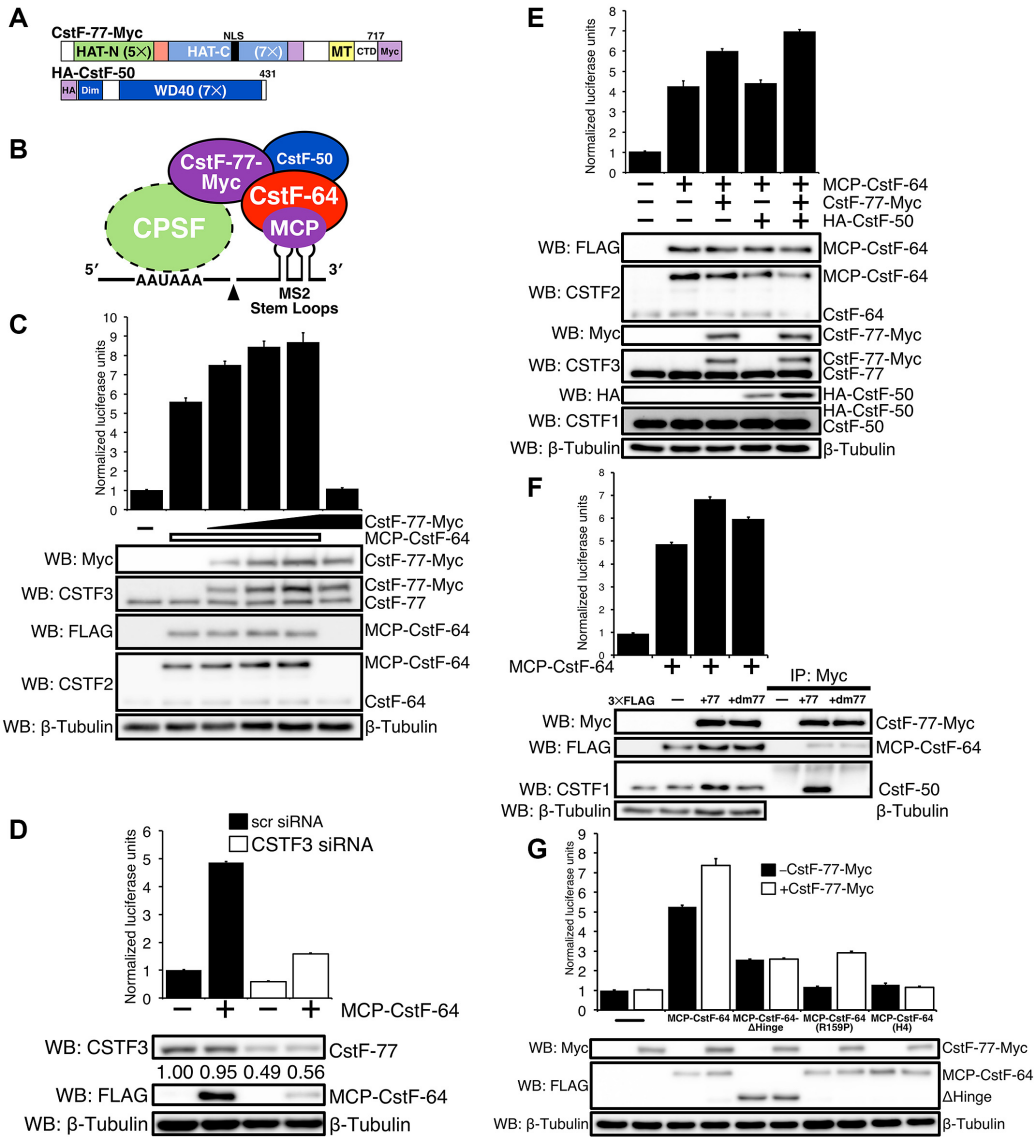
CstF-77 and CstF-50 enhance MCP-CstF-64-dependent cleavage and polyadenylation. (**A**) Schematic representation of CstF-77-Myc (CstF-77 with three Myc tags at its carboxy-terminal end) and HA-CstF-50 (CstF-50 with three hemagglutinin tags at its amino-terminal end). The position of the nuclear localization sequence (NLS), position and the number of the carboxy- and amino-terminal HATs, MT and the CTD of CstF-77 are shown. Similarly, the seven WD40 (WD40) repeats and the dimerization (Dim) domains are shown for CstF-50. (**B**) Cartoon of the interactions between MCP-CstF-64, CstF-77, CstF-50 and SL-Luc reporter construct. (**C**) Increased amounts of CstF-77-Myc increase C/P as measured by SLAP. Western blots with an antibody against Myc (WB: Myc), against CstF-77 (WB: CSTF3), against MCP-CstF-64 (WB: FLAG) or CstF-64 (WB: CSTF2) are shown. Antibodies against β-tubulin were used as a loading control. (**D**) Decreased CstF-77 reduces C/P as measured by SLAP. Hela cells were transfected with either a non-specific (scr) or CSTF3-specific siRNAs. Twelve hours after siRNA transfection, cells were transfected for SLAP. Western blots show the reduced expression of the endogenous CstF-77, MCP-CstF-64 (WB: FLAG). (**E**) HA-CstF-50 co-expressed with MCP-CstF-64 and CstF-77-Myc increases C/P as measured by SLAP. Western blots with the respective antibodies to verify expression of the exogenous proteins through their tags (WB: FLAG, Myc, HA) and antibodies to show relative expression to the endogenous proteins (WB: CSTF1, CSTF2, CSTF3). (**F**) Mutations in CstF-77-Myc that disrupt the interaction of CstF-77 with CstF-50 reduce C/P as measured by SLAP. Two-point mutations were made in CstF-77 (P584A, M589A, collectively called +dm77) that disrupt the interaction of CstF-77 with CstF-50. Immunoprecipitation with an antibody against Myc tag: no CstF-77, CstF-77-Myc (+77) and CstF-77-Myc-dm77 (+dm77). Western blots for Myc tag (WB: Myc), and endogenous CstF-50 (WB: CSTF1) and β-tubulin were presented. (**G**) Mutations in MCP-CstF-64 that disrupt the interaction of CstF-64 with CstF-77 decrease both SLAP and the stimulatory effect of CstF-77. MCP-CstF-64-ΔHinge lacks amino acids 96–216 from CstF-64. MCP-CstF-64 (R159P) is a point mutation in the Hinge domain that interrupts the interaction with CstF-77, MCP-CstF-64 (H4) is a series of point mutations in the fourth α-helix of the Hinge domain that interrupt interaction with CstF-77.

We wanted to test whether CstF-77 or CstF-50 would also affect C/P of the reporter gene. We cloned and expressed CstF-77 with a 3 × Myc epitope-tag at its carboxy-terminal end and CstF-50 with three hemagglutinin (HA) epitope tags at its amino-terminal end (Figures 2A and B). In the absence of MCP-CstF-64, addition of exogenous CstF-77-Myc did not result in a significant increase in luciferase expression compared to the control (Figure 2C). However, in the presence of MCP-CstF-64, addition of CstF-77-Myc led to an additional ∼50% increase of luciferase activity over MCP-CstF-64 alone. Thus, CstF-77 increases the production of luciferase from the SL-Luc construct through interactions with MCP-CstF-64, despite lack of a direct interaction of CstF-77 with the pre-mRNA. We call this increase of MCP-CstF-64-driven luciferase activity in the presence of CstF-77-Myc the ‘stimulatory effect.’

Next, we reduced endogenous CstF-77 using siRNAs (Figure 2D). In the absence of MCP-CstF-64, reduced expression of endogenous CstF-77 resulted in diminished luciferase production by more than half. This suggested that CstF-77 was important for C/P, even for the low expression levels of the SL-Luc construct in the absence of MCP-CstF-64. Similarly, reduced CstF-77 in the presence of MCP-CstF-64 resulted in a 3-fold decrease of luciferase activity. Interestingly, we noted that the expression of MCP-CstF-64 was decreased in cells in which CstF-77 was reduced (Figure 2D). This can be explained by lowered expression of the MCP-CstF-64 plasmid due to overall diminished C/P in the siRNA-transfected cells.

Expression of HA-tagged CstF-50 in the presence of MCP-CstF-64 did not increase C/P of the luciferase reporter (Figure 2E). Previous reports suggested that CstF-50 interacts with CstF-77 but not with CstF-64 and that all three subunits must be expressed simultaneously to make a functional CstF (23,41). In agreement, when we co-expressed all three proteins, we noticed a further increase of SLAP of ∼64% over MCP-CstF-64 alone and ∼16% over MCP-CstF-64 and CstF-77-Myc (Figure 2E), indicating enhancement of the stimulatory effect. We also noticed that HA-CstF-50 increased in HeLa cells in the presence of increased CstF-77-Myc. This might reflect the same co-translational regulation seen with CstF-64 (see above).

Yang *et al.* (41) identified the interacting surfaces between CstF-77 and CstF-50. Based on this report, we created a mutant in CstF-77-Myc (CstF-77-Myc-dm77, mutating both P584A and M589A) that abrogated the interaction with CstF-50 (Figure 2F). As predicted, CstF-77-Myc-dm77 does not interact with CstF-50 as assessed by co-immunoprecipitation (Figure 2F). In the presence of CstF-77-Myc-dm77, SLAP values were reduced compared to wild-type CstF-77-Myc, suggesting that CstF-77 must also interact directly with CstF-50 to participate in the stimulatory effect.

Finally, we explored interactions of the Hinge domain of CstF-64 with the MT domain of CstF-77 (21). For this experiment, we used two constructs that have mutations in interacting surfaces of the Hinge domain and the MT. MCP-CstF-64(R158P) has a single amino acid substitution that disrupts helix 3 of the Hinge (20,21); MCP-CstF-64(H4) has five mutations that disrupt helix 4 (21,46). As previously reported, CstF-64 from which the entire Hinge domain had been removed (MCP-CstF-64-ΔHinge) reduced SLAP by about 2.5 ± 0.07 NLUs (20). Addition of CstF-77-Myc to MCP-CstF-64-ΔHinge did not increase SLAP, corroborating the need for interactions between the MT and Hinge domain for CstF function. Similarly, transfection of MCP-CstF-64(R158P) and MCP-CstF-64(H4) into HeLa cells resulted in little luciferase expression above background (Figure 2G). However, co-transfection of CstF-77-Myc with MCP-CstF-64(R158P) showed an increase of luciferase activity, while co-transfection of CstF-77-Myc with MCP-CstF-64(H4) did not show such an increase (Figure 2G). This suggests that MCP-CstF-64(R158P), having a single disruption of the Hinge/MT interface, might retain some interaction with CstF-77, while MCP-CstF-64(H4) with five mutations disrupted that interaction completely.

### CstF-77 drives CstF-64 into the nucleus

**Figure 3.**
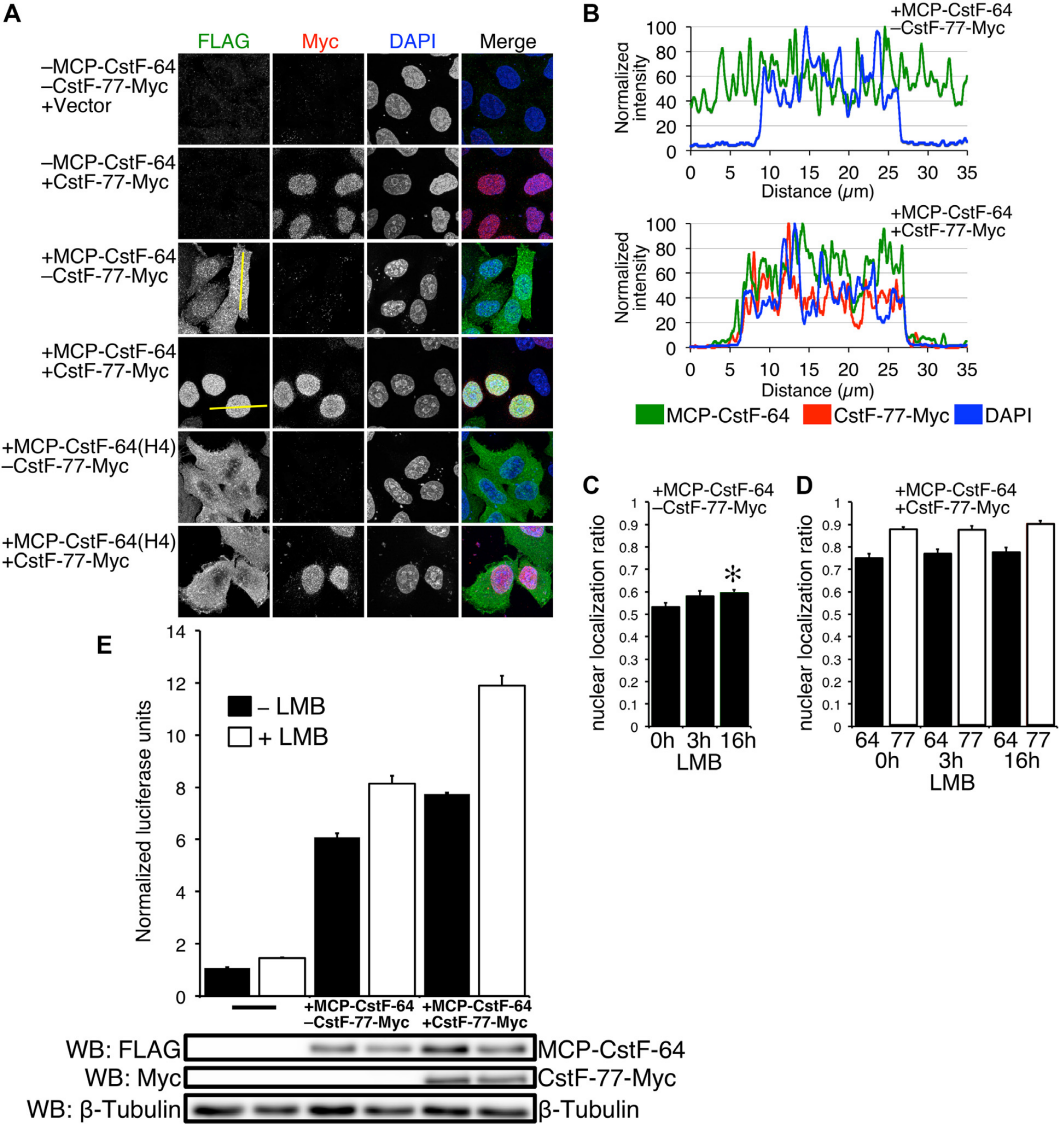
MCP-CstF-64 distributes equally between the cytoplasm and nucleus and does not shuttle between these cellular compartments. (**A**) Immunofluorescent confocal images of the described constructs stained with antibodies against the FLAG tag for MCP-CstF-64 (green) and Myc tag for CstF-77 (red). Cells were counterstained with DAPI to delineate the nucleus. Yellow lines through the cells indicate the position of the intensity profile of these cells shown in B. (**B**) Intensity signal profile of the cells shown in A. Left —a cell transfected only with MCP-CstF-64, right cell co-transfected with MCP-CstF-64 and CstF-77-Myc. (**C**) Quantification of the nuclear to cytoplasmic ratio of cells that were not treated with LMB (0 h), after 3 and 16 h of treatment (3 and 16 h, respectively). (**D**) Effect on addition of CstF-77-Myc on the nuclear to cytoplasm ratio of MCP-CstF-64 (black bars) with or without treatment with LMB at the indicated time points. Open bars—nuclear to cytoplasmic ratio for CstF-77-Myc. (**E**) LMB increases SLAP. A bar graph of cells with no additional MCP-CstF-64, with MCP-CstF-64 and additional CstF-77-Myc, with treatment for 16 h or no treatment. Representative western blots are shown to demonstrate the amount of exogenous proteins present in the assay.

Previously, it was shown that CstF-64 lacked a nuclear localization signal (NLS) and translocated into the nucleus because of its interaction with CstF-77, which possesses an NLS (20,46). Therefore, we hypothesized that at least part of the increase of C/P in SLAP samples co-transfected with CstF-77-Myc was due to an excess of CstF-77-Myc translocating MCP-CstF-64 into the nucleus. To test this, we examined subcellular localizations of MCP-CstF-64 or MCP-CstF-64(H4) with or without co-transfection of CstF-77-Myc (Figure 3A). In the absence of exogenous CstF-77-Myc, the majority of HeLa cells expressing MCP-CstF-64 showed a nearly equal distribution of signal between the cytoplasm and the nucleus (Figure 3B). Cells expressing less MCP-CstF-64 usually showed more nuclear localization than moderate or high expressing cells (Figure 3A). However, exogenous co-expression of CstF-77-Myc with MCP-CstF-64 resulted in near complete elimination of cytoplasmic MCP-CstF-64 (Figure 3A and B). On the other hand, the MCP-CstF-64(H4) mutant, which does not interact with CstF-77, showed cytoplasmic localization when co-expressed with CstF-77-Myc (Figure 3A). This supports the hypothesis that CstF-77 is the vehicle that drives CstF-64 into the nucleus and might be a limiting component of CstF function.

### CstF-64 and CstF-77 do not shuttle between cytoplasm and nucleus via the CRM/exportin 1 pathway

Many predominantly nuclear proteins shuttle between the cytoplasm and the nucleus (59,60). To determine whether CstF-64 shuttles between these two cellular compartments, we treated MCP-CstF-64-transfected cells with 20 nM leptomycin B (LMB), a potent and specific inhibitor of nuclear export mediated by CRM/exportin 1 protein (61–64). We then assessed the nuclear accumulation of MCP-CstF-64 by immunocytochemistry (Figure 3C). After 3 h of treatment, we did not see a statistically significant increase of nuclear localization for MCP-CstF-64 (Figure 3C). However, after 16 h of treatment, we observed a small (∼5%), but statistically significant increase in the nuclear localization of MCP-CstF-64. When we co-transfected MCP-CstF-64 and CstF-77-Myc, nuclear localization of MCP-CstF-64 increased by an additional 20% (Figure 3D). Treatment with LMB for either 3 or 16 h did not change the apparent nuclear localization of the MCP-CstF-64 protein when co-transfected with CstF-77-Myc (Figure 3D, black bars). Similarly, CstF-77-Myc nuclear localization did not change with the LMB treatment (Figure 3D, white bars). We conclude that neither CstF-77 nor CstF-64 are exported from the nucleus via the CRM/exportin 1 pathway.

We also measured C/P with the SLAP system after 16-h incubation with LMB, with or without co-expression of CstF-77-Myc (Figure 3E). HeLa cells expressing only MCP-CstF-64 and not treated with LMB had a SLAP value of about 6.10 ± 0.14 NLU (Figure 3E). Incubation with LMB for 16 h increased the SLAP value to 8.12 ± 0.30 NLU. Co-transfection with the CstF-77-Myc construct increased the normalized SLAP value as before. Treatment with LMB increased the SLAP values to 11.89 ± 0.39 NLU (Figure 3E), the highest value we were able to achieve in all of our SLAP experiments. We infer that some component of the polyadenylation machinery—but not CstF-64 or CstF-77—enhances C/P in HeLa cells when blocked from nuclear export.

### The CstF-64 RNA recognition motif is necessary for nuclear localization and the stimulatory effect by CstF-77

**Figure 4.**
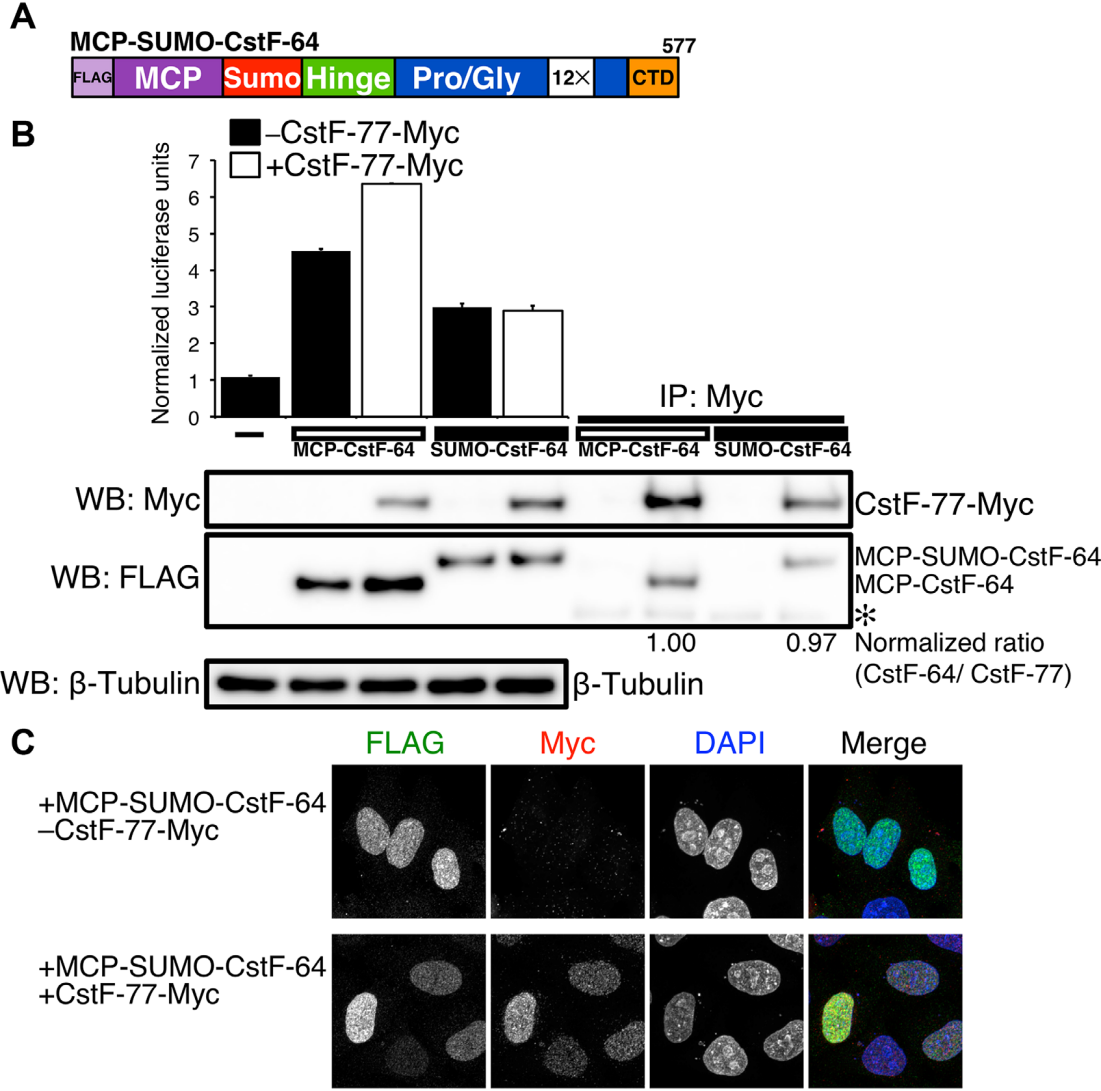
The RRM of CstF-64 contributes to SLAP. (**A**) Illustration of the MCP-SUMO-CstF-64 construct. The first 107 amino acids of the CstF-64 (RRM) is replaced by the SUMO domain (103 amino acids). (**B**) SLAP with the MCP-CstF-64 and MCP-SUMO-CstF-64 alone (black bars) and co-transfected with CstF-77-Myc (open bars). Western blots with antibody recognizing FLAG and Myc tags. On right, immunoprecipitation with an antibody against the Myc tag in the order of inputs: MCP-CstF-64 alone, MCP-CstF-64 co-transfected with CstF-77-Myc, MCP-SUMO-CstF-64 alone co-transfected with CstF-77-Myc. Numbers beneath the immunoprecipitations show the normalized ratio between MCP-CstF-64, MCP-SUMO-CstF-64 and CstF-77. (**C**) Immunohistochemistry on HeLa cells transfected with the MCP-SUMO-CstF-64 (green) alone and co-transfected with CstF-77-Myc (red). DNA in the nucleus was counterstained with DAPI.

Previously, we proposed that the RRM of CstF-64 played a role in RNA binding and possibly another role in C/P, since its deletion reduced MCP-mediated SLAP (20). Therefore, we wanted to examine functions for the RRM domain beyond recognition of the GU-rich sequence. CstF-64 lacking the RRM was poorly expressed in mammalian cells (reference 20 and unpublished observations). Therefore, to test its function, we replaced the first 107 amino acids of CstF-64 (encompassing the RRM) with a 103 amino acid domain from the SUMO protein (47,65) to create MCP-SUMO-CstF-64 (Figure 4A). Replacement of the RRM with the SUMO domain restored expression levels of CstF-64 (Figure 4B). SLAP indicated that MCP-SUMO-CstF-64 increased overall C/P above background but was reduced relative to full-length MCP-CstF-64 (Figure 4B). This suggests that the RRM of CstF-64, which is not required for RNA-binding in the SLAP system, affects C/P due to a non-RNA-binding mechanism.

More surprising to us was that co-expression of CstF-77-Myc with MCP-SUMO-CstF-64 did not stimulate SLAP (Figure 4B). Therefore, we wanted to determine whether addition of the SUMO domain to MCP-CstF-64 interfered with the interaction of CstF-64 with CstF-77. Immunoprecipitation of CstF-77-Myc demonstrated that MCP-SUMO-CstF-64 interacted with CstF-77-Myc to the same extent as did MCP-CstF-64 (Figure 4B).

MCP-SUMO-CstF-64 localized predominantly to the nucleus (Figure 4C), unlike the nearly equal distribution of MCP-CstF-64 between cytoplasm and nucleus (Figure 3A and B). Addition of CstF-77-Myc did not alter the localization of MCP-SUMO-CstF-64 (Figure 4C). This suggested the hypothesis that distribution of CstF-64 between cytoplasm and nucleus is mediated in part through RNA-binding by the RRM in addition to its interaction with CstF-77.

### Sites in the RRM of CstF-64 are necessary for stimulation of cleavage and polyadenylation by CstF-77 and nuclear localization

**Figure 5.**
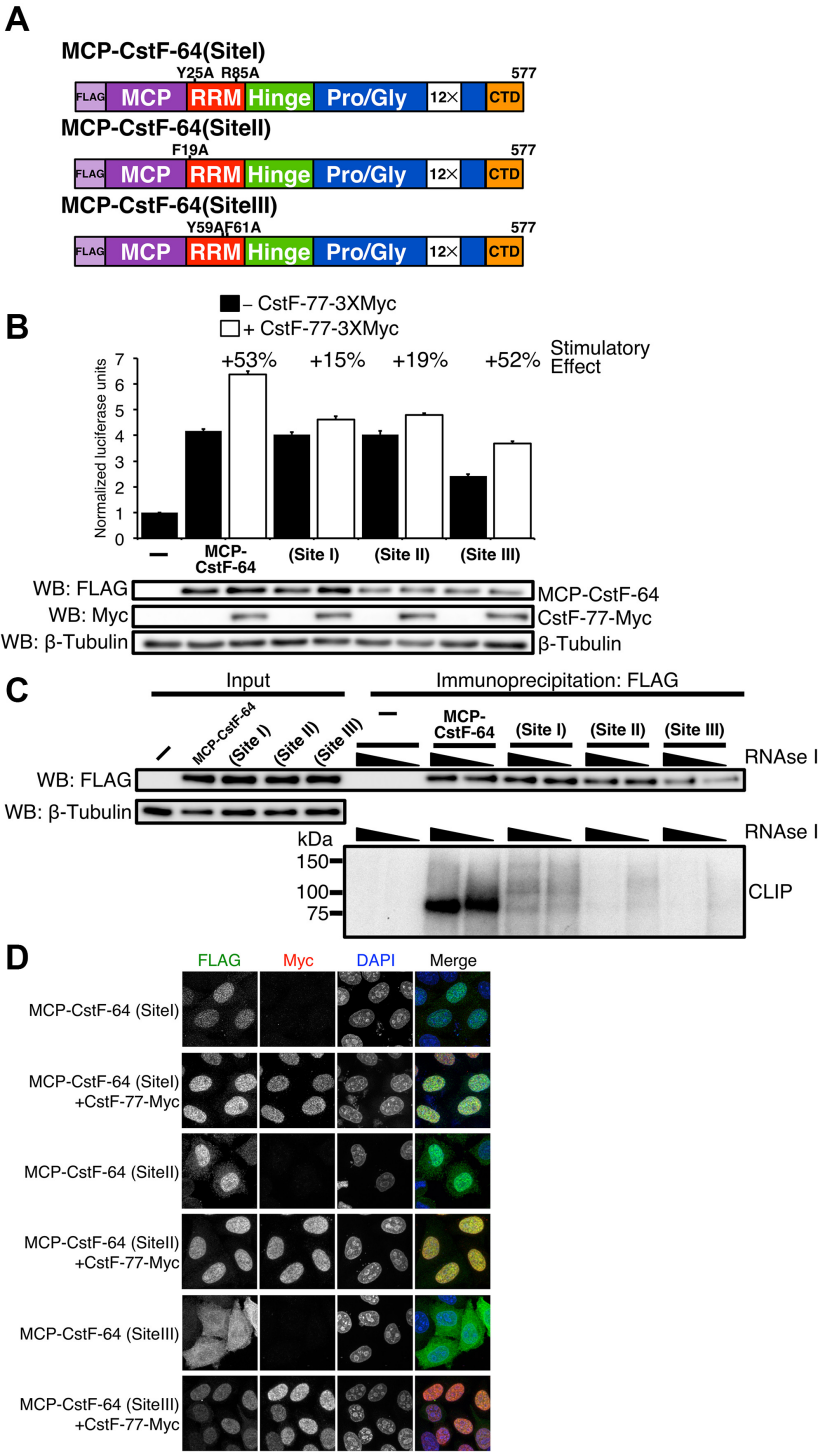
RNA binding of RRM is needed for the enhancement of SLAP provided by CstF-77. (**A**) Schematic illustration of point mutants in sites I, II and III of the RRM of MCP-CstF-64. (**B**) SLAP of the wild- type and I, II and III mutants of the RRM of CstF-64 alone and co-expressed with CstF-77-Myc. Western blots were performed to show expression of MCP-CstF-64 constructs and CstF-77-Myc. β-tubulin was used as a loading control. (**C**) CLIP experiment verifying that site I, II and III MCP-CstF-64 mutants do not bind or bind minimally to RNA (bottom). Western blot of the immunoprecipitated samples is also shown (top). (**D**) Immunohistochemistry staining of site I, II and III MCP-CstF-64 mutants. Site I and II localize to the nucleus when expressed alone (green). Site III demonstrates a cytoplasmic localization when expressed alone (green). All MCP-CstF-64 mutants locate to the nucleus when co-expressed with CstF-77-Myc (red).

Pancevac *et al.* (18) established three sites within the RRMs of CstF-64 and its yeast homolog RNA14 that mediate binding to GU-rich RNA sequences. To test whether RNA binding by CstF-64 is required for increased C/P mediated by CstF-77 in our SLAP assay, we introduced several point mutations into MCP-CstF-64 that abrogated binding of the endogenous RRM to RNA in each of these sites (Figure 5A and Supplementary Figure S1); these mutations did not affect MCP binding to MS2 sites in SL-Luc. SLAP values for mutations in site I or site II did not differ greatly from wild-type MCP-CstF-64 when expressed alone (Figure 5B). However, the site III mutant showed reduced normalized SLAP values. This suggested that, when divorced from their role in RNA binding, neither site I nor site II is necessary for C/P via CstF-64 in our reporter gene assay.

Interestingly, when site I and II mutants were co-expressed with CstF-77, we observed a reduced stimulatory effect of CstF-77 on SLAP as seen for wild-type MCP-CstF-64 (+15% and +19%, compared to +53%, Figure 5B). This suggested that sites I and II are necessary for the stimulatory effect of C/P by CstF-77. Co-expression of the site III mutant with CstF-77 showed stimulation of SLAP similar to that by wild-type MCP-CstF-64, suggesting that site III is not necessary for this effect. Together, these results suggest that CstF-77 plays an additional role in C/P beyond being the vehicle for transport of CstF-64 into the nucleus.

We created a double mutant of MCP-CstF-64 that contained mutations in site I and a pair of mutations in the Hinge (H4) domain of CstF-64. SLAP experiments with the double mutant showed only background values for SLAP with or without co-expression of CstF-77 (Supplementary Figure S2). These results suggested that the additional function of CstF-77 depended upon the interaction between the Hinge domain of CstF-64 and the MT of CstF-77. It also suggests that the majority (∼2/3) of the stimulatory effect of CstF-77 is due to the influence of CstF-77 on the RRM and not on the translocation of CstF-64 to the nucleus.

To confirm that the site I, II, and III mutants did not bind to RNA *in vivo*, we performed a crosslinking and immunoprecipitation (CLIP) experiment (50,66). HeLa cells transfected with the wild-type, site I, site II or site III mutant MCP-CstF-64 were exposed to UV light to crosslink RNA to bound proteins (Figure 5C). Wild-type MCP-CstF-64 labeled efficiently with radioactive [γ-^32^P]ATP, revealing a band above 75 kDa in the samples (Figure 5C, bottom), indicating RNA binding. However, none of the site I, II or III mutants showed meaningful amounts of labeling with ^32^P, suggesting that they interacted with RNAs only minimally in HeLa cells (Figure 5C). It also suggested that all three sites in the RRM need to be active for the complete function of the RRM.

Finally, we wanted to test whether the subcellular localization of site I, II or III MCP-CstF-64 mutants changed. Site I and II MCP-CstF-64 mutants expressed without CstF-77 showed exclusive nuclear localization (Figure 5D), different from the wild-type CstF-64 (Figure 3A). In contrast, the site III mutant showed more cytoplasmic localization than did the other two mutants (Figure 5D). When co-expressed with CstF-77-Myc, all mutants of MCP-CstF-64 showed predominantly nuclear localization (Figure 5D). These results suggest that the RNA binding provided by the site I and II is necessary for cytoplasmic localization of the CstF-64 under conditions of reduced amount of CstF-77, but that site III contributes less to that localization. Together, these results suggest that under conditions where CstF-64 exceeds CstF-77 (i) excess CstF-64 remains in the cytoplasm, and (ii) retention of CstF-64 in the cytoplasm is mediated by its binding to cytoplasmic RNAs. An increase in the amount of CstF-77 overcomes the cytoplasmic localization and moves CstF-64 to the nucleus.

### The carboxy-terminal domain of CstF-77 is important for cleavage and polyadenylation

**Figure 6.**
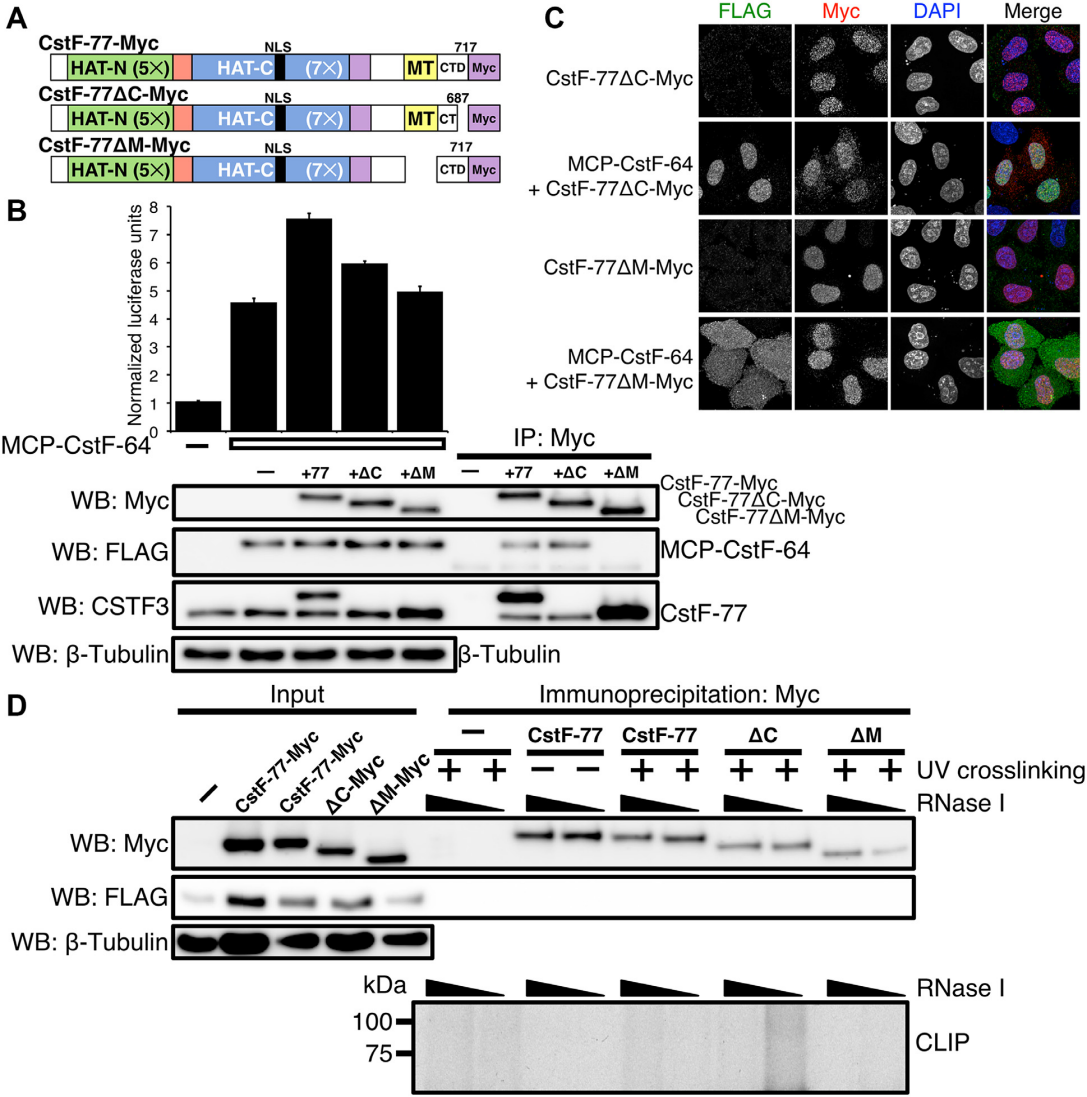
The CTD of CstF-77 is involved in modulating the binding affinity of the RRM of CstF-64. (**A**) Illustration of CstF-77-Myc deletion mutants: the last 30 amino acids of the carboxy terminal domain (amino acids deleted Δ688–717, CstF-77ΔC-Myc), and MT Δ607–664, CstF-77ΔM-Myc). (**B**) SLAP of the wild-type and CstF-77-Myc deletion mutants. Western blots to verify the expression of the proteins: antibodies against Myc tag to detect CstF-77-Myc construct, FLAG to detect MCP-CstF-64 and against CSTF3 to detect endogenous and exogenous CstF-77 protein. Note that the antibody against CSTF3 is raised against a peptide within the last 30 amino acids of the CstF-77 and therefore does not recognize the CstF-77ΔC-Myc construct. (**C**) Immunohistochemistry staining of the CstF-77-Myc deletion mutants (red) alone or co-transfected with MCP-CstF-64 (green). DNA in the nucleus is counterstained with DAPI. (D) CLIP (bottom) of the CstF-77-Myc deletion mutants. UV-crosslinking or the lack of it is indicated. The increasing amount of RNase I is also indicated. Amount of the proteins in the input is shown for MCP-CstF-64 (WB: FLAG), wild-type CstF-77-Myc and deletion mutants (WB: Myc). β-tubulin was used as a loading control. Note that in the conditions of CLIP using RIPA buffer, MCP-CstF-64 does not co-immunoprecipitated with CstF-77-Myc.

**Figure 7.**
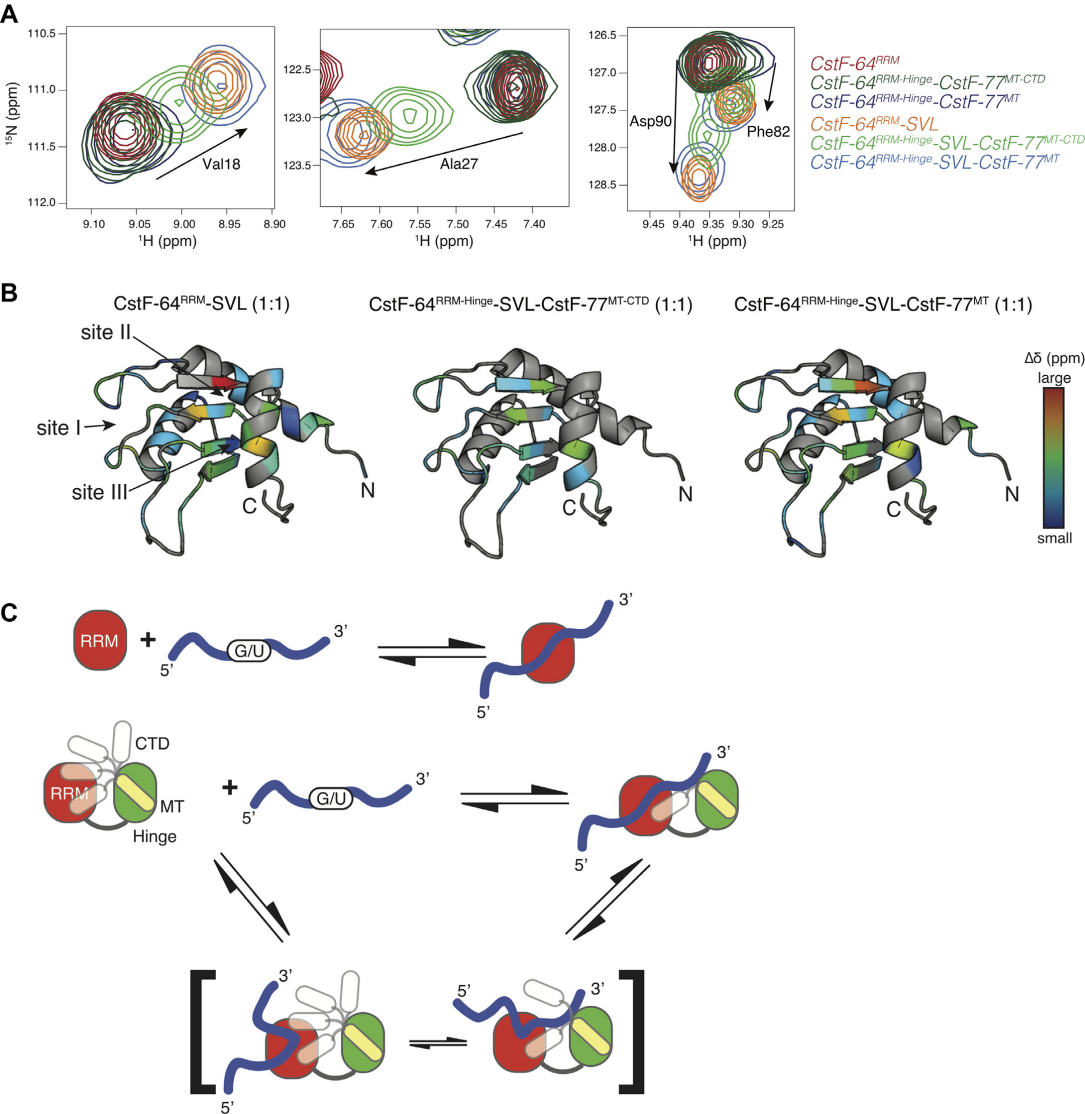
The CTD of CstF-77 perturbs CstF-64^RRM^-RNA binding. (**A**) Overlay of 2D ^15^N-^1^H HSQC spectra for four different residues experiencing CSPs upon the addition of SVL RNA. Red, dark green and dark blue contours represent the apo form of CstF-64^RRM^, CstF-64^RRM-Hinge^-CstF-77^MT-CTD^ and CstF-64^RRM-Hinge^-CstF-77^MT^, respectively, whereas the orange, light green and light blue contours represent the 1:1 protein—SVL RNA complexes for CstF-64^RRM^, CstF-64^RRM-Hinge^-CstF-77^MT-CTD^ and CstF-64^RRM-Hinge^-CstF-77^MT^, respectively. (**B**) Ribbon diagrams of the CstF-64^RRM^ structure depicting the changes in ^15^N-^1^H chemical shift upon titration of SVL RNA into CstF-64^RRM^ (left), CstF-64^RRM-Hinge^-CstF-77^MT-CTD^ (middle) and CstF-64^RRM-Hinge^-CstF-77^MT^ (right), respectively. The dark blue-to-red gradient represents backbone amide groups that experience a small-to-large weighted, averaged CSP (0.037–0.24 ppm) (55). Arrows point to the three RNA binding sites (site I, II, III) described by Taylor and co-workers (18). (**C**) Model of illustrating a possible effect on the CTD of CstF-77 on RNA binding. The *top* scheme shows the simple two-state model employed by CstF-64^RRM^ and CstF-64^RRM-Hinge^-CstF-77^MT^ complex. The *bottom* scheme includes a dynamic complex where the CTD of CstF-77 can adopt multiple conformations, which can occlude binding to sites within the RRM of CstF-64.

We wondered which domains of CstF-77 might influence cleavage and polyadenylation through interactions with the RRM of CstF-64. The C-terminal domain of CstF-77 (CTD) adjacent to the MT interaction domain is predicted to form an α-helix that is conserved in metazoans (Supplementary Figure S3) but absent in yeast (21). Because of its proximity to the domain by which CstF-77 interacts with CstF-64, such a structure might contribute to the influence of CstF-77 on CstF-64-mediated C/P. To test this, we deleted the last thirty amino acids (688–717) of the CTD of CstF-77-Myc and performed SLAP (CstF-77ΔC-Myc, Figures 6 and 7A). We also deleted the MT (amino acids 607–664; CstF-77ΔM-Myc) as a control. Co-expression of CstF-77-Myc with MCP-CstF-64 increased luciferase expression as before with the stimulatory effect (Figure 6B). On the other hand, co-expression of CstF-77ΔM-Myc with MCP-CstF-64 did not show any differences from the SLAP value obtained from MCP-CstF-64 alone (Figure 6B). This confirms that the CstF-77 MT is necessary for the interaction between CstF-77 and CstF-64 in this assay.

Immunoprecipitation of CstF-77-Myc with an anti-Myc antibody also co-precipitated endogenous CstF-77 (Figure 6B), indicating that at least two copies of CstF-77 are in the CstF complex. To our knowledge, this is the first *in vivo* demonstration that CstF functions as a hexamer (dimer of trimers), as has been suggested previously by structural studies (37,38). Deletion of the MT domain from CstF-77 (CstF-77ΔM-Myc) abrogates this effect (Figure 6B).

Co-expression of CstF-77ΔC-Myc with MCP-CstF-64 reduced the stimulatory effect by about half (Figure 6B), suggesting that the predicted C-terminal α-helix of CstF-77 participates in the stimulatory C/P effect. We interpret the incomplete reduction to reflect the likelihood that CstF acts as a hexamer through a CstF-77 heterodimeric CstF-77/CstF-77ΔC-Myc interaction, and thus has partial activity. CstF-77ΔC-Myc was not detected because the anti-CstF-77 antibody is directed to an epitope at the last 30 amino acids of CstF-77. We conclude that the CTD of CstF-77 influences both C/P and the stimulatory effect as mediated by CstF-64.

We also co-expressed MCP-CstF-64(Site I) with the CstF-77-Myc mutants lacking the last 30 amino acids or the MT. Co-expression of MCP-CstF-64 (Site I), which interferes with RNA binding, with either CstF-77ΔC-Myc or CstF-77ΔM-Myc completely eliminated the enhancement contributed by CstF-77 to C/P (Supplementary Figures S2, 4 and 5). This agrees with the observation that the stimulatory effect of CstF-77 requires amino acids involved in RNA binding by CstF-64 (Figure 5B).

Immunofluorescent visualization showed that the majority of CstF-77ΔC-Myc and CstF-77ΔM-Myc was localized to the nucleus with or without MCP-CstF-64. However, we observed a small fraction of CstF-77ΔC-Myc localized to small speckles in the cytoplasm (Figure 6C). This small fraction did not co-localize with MCP-CstF-64 (Figure 6C). Removal of the MT from CstF-77 did not influence the localization of CstF-77, but co-expressed MCP-CstF-64 was localized to the cytoplasm. This further supports that CstF-77 plays a role as the vehicle to transport CstF into the nucleus.

### CstF-77 does not bind RNA

In Arabidopsis, CstF-77 binds RNA (67) and a survey of RNA-binding proteins in human cells (68) suggested that 12 amino acids (698–709) of CstF-77 potentially bind RNA. Therefore, one possible explanation for the stimulatory effect is that the C-terminus of CstF-77 interacts directly with pre-mRNA during C/P to enhance RNA binding or specificity. To test whether CstF-77 binds RNA during C/P, we performed a UV-crosslinking (CLIP) experiment. Hela cells were transfected with the various CstF-77-Myc constructs together with MCP-CstF-64, subjected to crosslinking with UV light, RNAs were labeled with ^32^P, and the radioactively labeled RNA-CstF-77-Myc proteins complexes were resolved on SDS-PAGE. Contrary to the report of Castillo *et al.* (68), we did not observe the interaction between CstF-77-Myc and RNA (Figure 6D) under conditions that readily identified interactions between CstF-64 and RNA (Figure 5C). This indicates that CstF-77 does not bind RNA directly but instead stimulates C/P via a different interaction with the CstF-64 RRM.

### Solution NMR spectroscopy demonstrates that the last thirty amino acids of CstF-77 alter the binding to RNA

Co-expression of CstF-64 and CstF-77 is required to form a stable complex (21,69). We designed two bicistronic constructs that contained either the RRM-Hinge domain (amino acids 1–195) of CstF-64 and the MT-CTD (amino acids 607–717, CstF-64^RRM-Hinge^-CstF-77^MT-CTD^) or the RRM-Hinge domains of CstF-64 followed by the MT-CTD with the last 30 amino acids removed (CstF-64^RRM-Hinge^-CstF-77^MT^). For comparison, we also prepared the RRM (amino acids 1–107) of CstF-64 (17). All proteins were ^15^N-labeled, purified and the two-dimensional ^1^H-^15^N HSQC spectra of the three proteins was recorded (Supplementary Figure S5). The spectrum of the RRM matches previously assigned NMR peaks (16,17). The overlay of the HSQC spectra showed that the RRM peaks for the CstF-64^RRM-Hinge^-CstF-77^MT-CTD^ and CstF-64^RRM-Hinge^-CstF-77^MT^ complexes were at the same positions as for the isolated RRM. This suggests that the RRM is a well-defined structural entity with a similar conformational environment within the two complexes. This environment was only minimally perturbed by the presence of the Hinge domain of CstF-64 or the MT and the CTD of CstF-77 (Supplementary Figure S5).

By comparing the ^1^H-^15^N HSQC spectra of CstF-64^RRM-Hinge^-CstF-77^MT-CTD^ complex and CstF-64^RRM-Hinge^-CstF-77^MT^, we were able to identify peaks for 28 of the 30 amino acids missing in the CstF-64^RRM-Hinge^-CstF-77^MT^ complex (i.e. peaks belonging to the CTD of CstF-77; asterisks in Supplementary Figure S5). Two prolines in the CTD cannot be identified using ^1^H-^15^N HSQC.

Next, we titrated the samples with increasing amounts of an RNA oligonucleotide derived from the SV40 late transcription unit (SVL). We chose the SVL pre-mRNA because it is a strong viral RNA substrate whose C/P has been extensively studied in conjunction with the CstF complex (14,40,70). None of the peaks identified as part of the last 30 amino acids of CstF-77 in the CstF-64^RRM-Hinge^-CstF-77^MT-CTD^ complex showed significant ^1^H-^15^N chemical shift perturbations (CSPs) upon reaching 1:1 molar ratio with the SVL RNA (e.g. boxed peaks in Supplementary Figure S6, middle), indicating that their chemical environment was not altered upon RNA binding. This result further confirmed that the CTD of CstF-77 does not actively participate in RNA binding *in vitro* (Figure 6D).

Upon SVL RNA titration with the CstF-64^RRM-Hinge^-CstF-77^MT-CTD^ complex, we observed that several peaks in the HSQC spectra associated with the RRM moved, consistent with RNA binding to the RRM. RNA titration with the isolated RRM and the CstF-64^RRM-Hinge^-CstF-77^MT^ complex resulted in CSPs that were similar in amplitude and direction (see amino acid residues Val18, Ala27, Phe82 and Asp90, Figures 7A and Supplementary Figure S6), indicating that the structure of the bound state was similar for these complexes. On the other hand, CSPs observed for the CstF-64^RRM-Hinge^–CstF-77^MT-CTD^ complex upon SVL RNA binding were smaller in magnitude, but along the same direction (light green contours in Figure 7A, middle), demonstrating a difference in RNA binding for the CstF-64^RRM-Hinge^-CstF-77^MT-CTD^ complex. In all cases, the CSPs demonstrated that the SVL RNA still interacted with sites I, II, and III within the RRM but that the presence of the last 30 amino acids of CstF-77 alters SVL RNA binding in this context (Figure 7B).

Finally, the CSPs upon SVL RNA titration were used to calculate binding affinities (*K*_d_) for each protein-RNA complex (Supplementary Figure S6). During the titration of a protein-ligand complex, chemical shifts of nuclei that are at the binding interface are perturbed as they experience different chemical environments. These perturbations can be related to three main exchange regimes defined by two parameters: the exchange rate of the complex formation (*k*_ex_ = *k*_on_[RNA] + *k*_off_) and the difference in resonance frequency of the free and bound states (Δ*δ* = *δ*_free_ - *δ*_bound_) (71). Analysis of RNA-induced perturbations to RRM resonances that were in the fast exchange regime resulted in a *K*_d_ of 0.7 ± 0.4 μM for the isolated RRM–RNA complex. This value for the isolated CstF-64 RRM is in general agreement with earlier studies (41,72,73).

Inclusion of the Hinge domain of CstF-64 and the MT of CstF-77 slightly reduced the affinity of the CstF-64^RRM-Hinge^–CstF-77^MT^ complex for RNA to a *K*_d_ of 3.5 ± 1.0 μM (Supplementary Figure S6). We also obtained a *K*_d_ of 3.3 ± 2.3 μM for the SVL RNA binding to the CstF-64^RRM-Hinge^–CstF-77^MT-CTD^ complex, a value similar to the construct lacking the CTD. Note however that the difference in magnitude for the CSPs upon fully binding the SVL RNA leads to different exchange regimes: Ala27, Leu47 and Tyr49 are in the intermediate exchange regime for isolated RRM and CstF-64^RRM-Hinge^–CstF-77^MT^ complex; whereas, the smaller CSPs resulted in fast exchange for these residues in the titration of the CstF-64^RRM-Hinge^–CstF-77^MT-CTD^ complex (Supplementary Figure S6). This suggests that the last 30 amino acids of CstF-77 alter the RNA binding kinetics or mechanism for the RRM of CstF-64.

## DISCUSSION

### The CstF complex must be available in the nucleus for cleavage and polyadenylation

The tripartite cleavage stimulation complex (CstF) was the first polyadenylation factor to be structurally characterized (40,74). While the CPSF performs many mechanical functions of C/P, CstF appears to serve as the regulatory subunit to control direct and alternative C/P (24,33,46,58). While it has been accepted that CstF-64 is important for C/P by recognizing and binding to the GU-rich downstream sequence element (14), far less is known about the functional role of CstF-77 in nuclear C/P (22,69,75). Cytoplasmic functions of CstF-77 in translation are known (43), and these might be reflected in our localization of a portion of CstF-77-Myc in cytoplasmic speckles (Figures 3–6). However, the open questions about CstF-77 function led us to use the SLAP system to explore interactive roles of CstF-64 and CstF-77 in C/P.

Our results indicate a direct link between the abundance of the CstF complex and increased nuclear C/P. Specifically, we show that all subunits of the complex, CstF-50, CstF-64 and CstF-77, contribute to the enhancement of SLAP (Figures 1 and 2), supporting previous *in vitro* studies (41). These results suggest that the abundance of the CstF complex is limiting for some processes in C/P, thus signaling a role in control of alternative polyadenylation. For example, in cancer cells, which often show increased CstF-64, mRNA levels of genes with a single C/P site might be upregulated due to more efficient 3′ end processing (7,76–78). Similarly, increased CstF-77/CSTF3 has been associated with alternative 3′ end processing in cancer (79,80).

### CstF-77 is the vehicle transporting the CstF complex into the nucleus

Our initial hypothesis was that the only function of CstF-77 was to transport CstF to the nucleus while acting as a scaffold to connect CstF-64 and CstF-50 to each other (20,23,41,46) and to CPSF-160 (42). Our results support this hypothesis, but suggest it is not complete. Immunohistochemical staining of HeLa cells transfected with MCP-CstF-64 showed that it was distributed relatively homogeneously throughout the cells (Figure 3). This homogeneous distribution of CstF-64 was disrupted, however, when CstF-64 was co-expressed with CstF-77; upon co-expression, up to 75% of CstF-64 was localized to the nucleus (Figure 3). This suggests that there is a limited amount of CstF-77 within cells that is not sufficient to support the increased expression of the exogenously expressed MCP-CstF-64.

### RNA binding is required for CstF-64 retention in the cytoplasm

Surprisingly, mutations that destroyed RNA recognition of CstF-64 were sufficient to completely localize the protein to the nucleus (Figures 4 and 5), suggesting a role for RNA binding in CstF-64 localization. On the model of the human poly(A)-binding protein (81), we propose that, in the absence of nuclear localization through CstF-77, CstF-64 interactions with cytoplasmic RNAs are sufficient to retain a portion of it in the cytoplasm.

Increased cytoplasmic localization of CstF-64 was also reported during infection of human fibroblasts with human cytomegalovirus (82). This implies that cytoplasmic localization of CstF-64 might be a part of the normal cellular response to viral infections, for example to limit the ability of the virus to process its RNA. Similarly, limited amounts of CstF-64 might be implicated in a function separate from the cleavage and polyadenylation as reported for CstF-77, for example as a part of the masking complex (43).

### Neither CstF-64 nor CstF-77 shuttles between the cytoplasm and the nucleus during cleavage and polyadenylation

Because a substantial fraction of CstF-64 was found in the cytoplasm (Figure 3), we asked whether CstF or its individual components could shuttle between the nucleus and cytoplasm. Treatment of HeLa cells with LMB, an inhibitor of the CRM1/exportin 1-mediated nuclear export pathway, did not alter the partitioning of either CstF-64 or CstF-77 between nucleus and cytoplasm over the span of 3 h (Figure 3), the typical time course for nuclear-cytoplasmic shuttling (61–64). Longer treatments (16 h) only slightly increased CstF-64 retention in the nucleus, but significantly increased C/P as assessed by SLAP (Figure 3E). Such incomplete shuttling of an otherwise nuclear protein has been described previously (83). The unidirectional movement of CstF-64 and CstF-77 suggests that, once formed, the CstF complex performs multiple cycles of cleavage and polyadenylation in the nucleus before being inactivated and degraded. This further implies that CstF is stably associated with the CTD of pol II and may not dissociate from it during transcriptional re-initiation (84,85).

### CstF-77 regulates cleavage and polyadenylation through interactions with CstF-64 that modify its affinity for RNA substrates

Mutation of amino acids in the CstF-64 RRM that eliminate its ability to bind RNA offers interesting insights into how CstF-77 affects the function of CstF-64. Although, we initially thought that the SLAP system was independent of the RNA-binding function of the RRM (because RNA-binding was mediated through the MCP domain, reference 20), we now understand that the RRM contributes to C/P specifically when increased amounts of CstF-77 protein are available (Figure 2). This suggests that CstF-77 alters the binding interaction of the RRM for RNA either through a direct involvement in RNA binding, by modulating the affinity of the RRM for RNA, or by altering the mode of RNA binding of the RRM of CstF-64. Our CLIP and NMR experiments eliminated a direct involvement of CstF-77 in RNA binding (Figures 6 and Supplementary Figure 5), increasing the likelihood that CstF-77 acts indirectly on C/P, possibly through modulating the kinetics of CstF-64 binding to the RNA substrate (i.e. altering the mode of binding).

### The carboxy-terminal domain of CstF-77 alters the recognition of RNA through the RRM of CstF-64

A recent study measured RNA binding of the CstF-complex for RNA and suggested that CstF-77 was involved in increasing the affinity of CstF-64 for RNA (41). This is consistent with our *in vivo* findings (Figure 5 and Supplementary Figure S2). Our SLAP data suggest that the C-terminal domain of CstF-77 alters recognition of RNA mediated through the RRM of CstF-64 (Figure 6). Yet, our structural data demonstrate that neither the MT nor the CTD of CstF-77 interact directly with the RRM of CstF-64: most RRM peaks of the complex appear at the same chemical shifts as observed for the isolated RRM (Supplementary Figure S5). Instead, the largest chemical shift perturbations in the CstF-64 RRM occur at its C-terminal end adjacent to the Hinge. We interpret these NMR data to suggest that the effect of the CTD of CstF-77 is on the CstF-64 ^RRM-Hinge^–CstF-77 ^MT-CTD^ complex, though not through a direct interaction with the RRM. The effect could be allosteric, through interactions with the CstF-64 Hinge, or steric, by occupying the space around the RRM that is needed for the RNA–RRM interaction (Figure 7C).

The addition of the Hinge domain of CstF-64 and MT and CTD of CstF-77 altered RNA binding by the RRM (Figure 7 and Supplementary Figure S6). Surprisingly, we noted no significant differences in *K*_d_s for SVL RNA binding to the isolated RRM or to the CstF-64^RRM-Hinge^–CstF-77^MT-CTD^ and CstF-64^RRM-Hinge^–CstF-77^MT^ complexes. Nevertheless, we observed that the presence of the CTD shifted the end point (i.e. the final peak position) of the titration to ∼60–70% of the final position for RRM alone (Figure 7 and Supplementary Figure S6), implying that the presence of the CstF-77 CTD in the complex alters the RNA binding reaction by CstF-64. It has been shown that RNA binding by the RRM can be modeled from a simple binding equilibrium (Figure 7C, top) (18), which our RNA titration data supports. However, the presence of the CTD seems to alter the mode of RNA binding for the CstF-64^RRM-Hinge^–CstF-77^MT-CTD^ complex such that it is no longer a simple two state equilibrium, an example of which is illustrated in Figure 7C. The presence of a third (or more) state(s)—where the RNA is only partially bound to either sites I, II or III—could lead to the smaller magnitude chemical shift perturbations we observed in SVL RNA titration (Figure 7C). However the CTD of CstF-77 exerts its effect on RNA binding to the CstF-64^RRM-Hinge^–CstF-77^MT-CTD^ complex, it is an indirect effect as our *in vivo* RNA binding data and the NMR titration spectra for the CstF-64^RRM-Hinge^–CstF-77^MT-CTD^ complex showed no direct interaction of the CTD of CstF-77 with RNA (Figure 6 and Supplementary Figure S6).

We propose a model in which the last 30 amino acids of CstF-77 have opposing effects in the cytoplasm and nucleus. In the cytoplasm, the CTD of CstF-77 blocks the interaction of the cytoplasmic RNAs with the RRM of CstF-64. In the nucleus, under the influence of the CPSF complex or other proteins involved in C/P, the last 30 amino acids of CstF-77 experience conformational changes that lead to stabilization of the RNA binding surfaces and change the mode of binding of the RRM of CstF-64.

## Supplementary Material

Supplementary Data
