# Supplementary material for: The structural basis of CstF-77 modulation of cleavage and polyadenylation through stimulation of CstF-64 activity
Source: Nucleic Acids Res. 2018 Sep 26;46(22):12022–39. doi: 10.1093/nar/gky862 (PMC6294498; doi:10.1093/nar/gky862)
Supplement: Supplementary Data [file gky862_supplemental_files.pdf]

Supplemental Figure 1

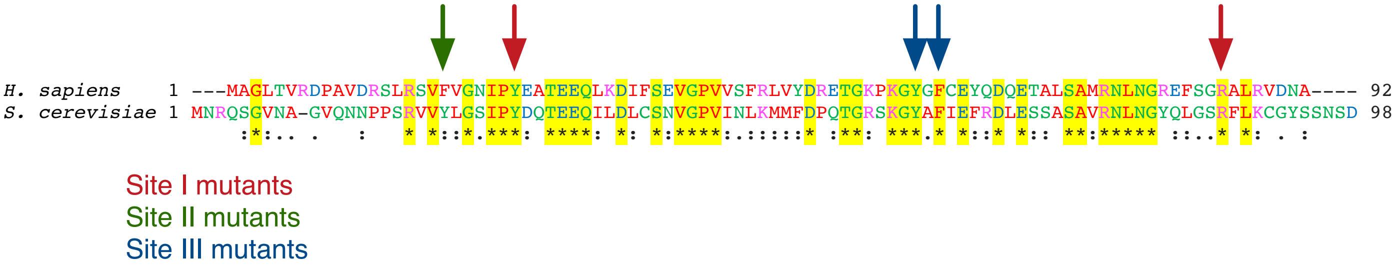

Figure S1. Alignment of the primary protein sequence of the RRM of CstF-64 (human) and Rna15p (yeast), related to Figure 5. The first ninety-two amino acid residues from human CSTF2 protein (accession number: NP_001316.1) and amino acids one to ninety-eight from yeast Rna15p protein (accession number: NP_011471.1), homolog of CstF-64 were aligned using Clustal Omega (Li et al. *Nucleic Acids Res* **43,** W580–584, 2015). The identical amino acids in both proteins are highlighted in yellow. An "*" (asterisk) indicates amino acids, which are conserved; a ":" (colon) indicates amino acids with a score greater than 0.5 in the Clustal Omega matrix; and a "." (period) indicates amino acids with a score smaller or equal to 0.5 the Clustal Omega matrix. Indicated amino acids for Site I (Y25, R85), II (F19), and III (Y59, F61) were mutated to alanines.

Supplemental Figure 2

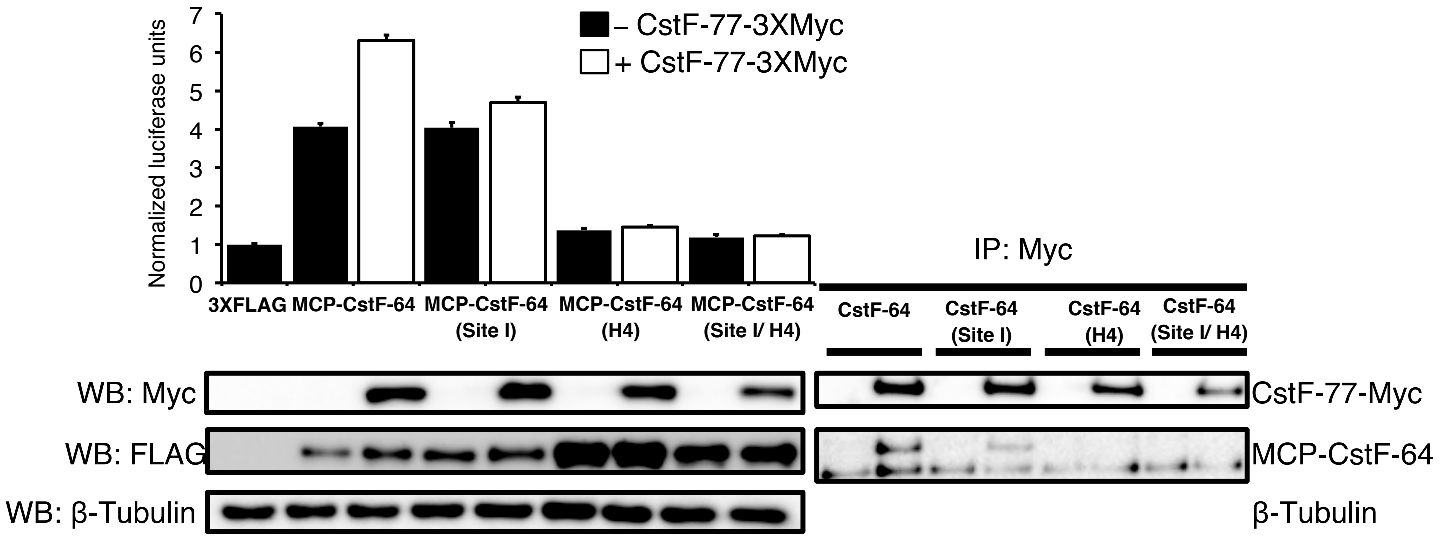

Figure S2. The interaction between the hinge of CstF-64 and the monkeytail of CstF-77 is needed for the stimulatory effect of CstF-77, related to Figure 5. A double mutant containing the H4 and Site I mutations in CstF-64 does not provide a stimulatory effect because of CstF-77. Normalized SLAP of the wild type, Site I, H4 and Site I/H4 double mutants of CstF-64 alone and co-expressed with CstF-77-Myc. Western blots were performed to show expression of MCP-CstF-64 constructs (WB: FLAG) and CstF-77-Myc (WB: Myc). β-tubulin was used as a loading control. Immunoprecipitation with an antibody against Myc tag: vector plasmid, followed by CstF-77-Myc combined with MCP-CstF-64, MCP-CstF-64 (site I), MCP-CstF-64 (H4), and MCP-CstF-64 (Site I/H4) double mutant.

Supplemental Figure 3

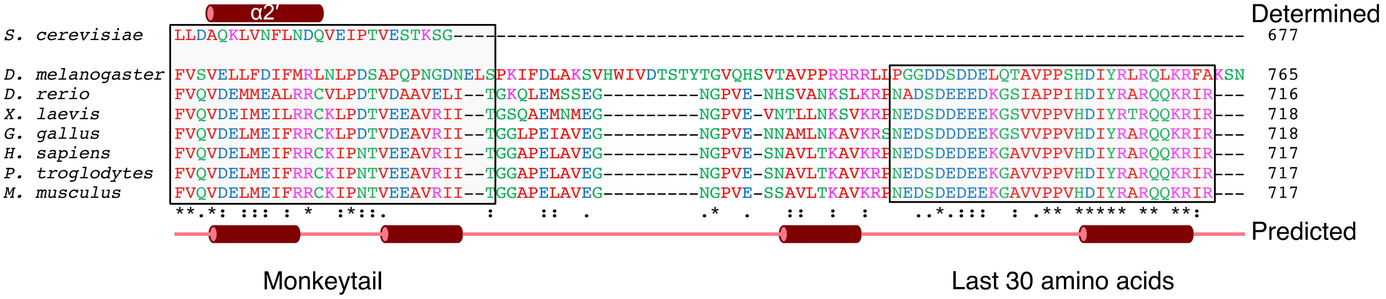

Figure S3. Comparison of the last eighty-five amino acid residues from human CstF-77, related to Figures 6 and 7. Amino acids 633–717 from human (*H. sapiens*, accession number: NP_001317.1), 666–733 from fruit fly (*D. melanogaster*, accession number: NP_001015241), 632–715 from zebrafish (*D. rerio*, accession number: NP_998218), 634–718 (*X. tropicalis*, accession number: NP_989162), 634–718 (*G. gallus*, accession number: NP_001012586.1), 633–717 chimpanzee (*P. troglodytes*, accession number: XP_508355.2), 633–717 mouse (*M. musculus*, accession number: NP_663504.1) were aligned using Clustal Omega algorithm. An "*" (asterisk) indicates amino acids that are conserved; a ":" (colon) indicates amino acids with a score greater than 0.5 in the Clustal Omega matrix; and a "." (period) indicates amino acids with a score smaller or equal to 0.5 the Clustal Omega matrix. The sequence of yeast (*S. cerevisiae*) Rna14p, homolog of CstF-77 is aligned as presented in Figure 3B from Moreno-Morcillo et al. (*Structure* **19,** 534–545, 2011). On the top the 2 helix (determined) is according yeast structure between the monkeytail of Rna14p (CstF-77) and hinge domain of Rna15p (CstF-64). At the bottom predicted secondary structure for the last eighty-five amino acids of human CstF-77 using PSIPRED Protein Analysis Workbench (Buchan et al. *Nucleic Acids Res* **41,** W349–357, 2013). Half of the monkeytail that is present and the last thirty amino acids of CstF-77 are boxed.

Supplemental Figure 4A

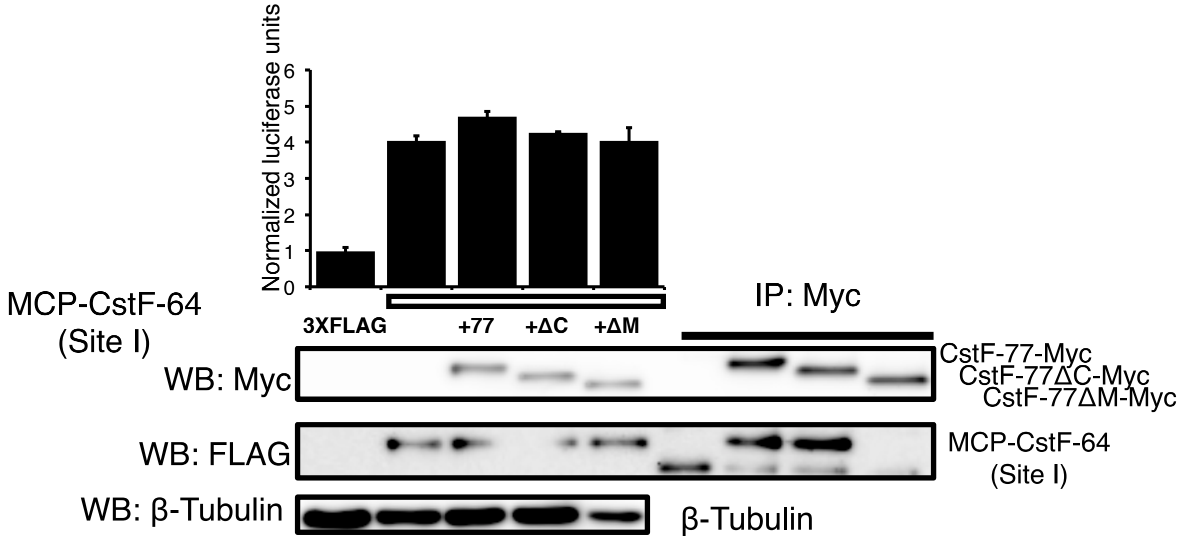

Supplemental Figure 4B

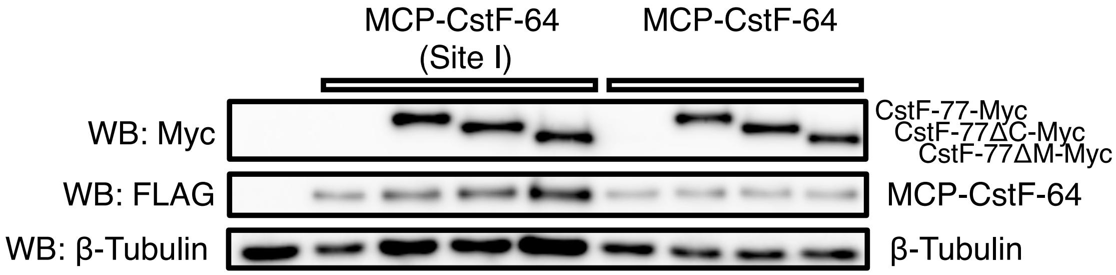

Figure S4. Co-expression of MCP-CstF-64 (Site I) with CstF-77-Myc, CstF-77C-Myc, CstF-77M-Myc completely eliminates the stimulatory effect of CstF-77 in SLAP, related to Figure 6. **(A)** Normalized SLAP of the MCP-CstF-64 (Site I) mutant of the RRM of CstF-64 co-expressed with vector, CstF-77-Myc, CstF-77ΔC-Myc, CstF-77ΔM-Myc. Expression of the MCP-CstF-64 (Site I) is shown by Western blot with FLAG antibody (WB: FLAG). Expression of the CstF-77-Myc, CstF-77ΔC-Myc, CstF-77ΔM-Myc is shown by Western blot with anti-Myc antibody (WB: Myc). -tubulin was used as a loading control. MCP-CstF-64 (site I) mutant interacts with CstF-77ΔC-Myc as shown by co-immunoprecipitation with an antibody against the Myc tag. **(B)** MCP-CstF-64 and MCP-CstF-64 (Site I) are expressed in relatively equal amounts. Expression of MCP-CstF-64 (Site I) and MCP-CstF-64 (WB: FLAG) without or with co-expression of CstF-77-Myc, CstF-77ΔC-Myc, CstF-77ΔM-Myc (WB: Myc) and β-tubulin was used as a loading control.

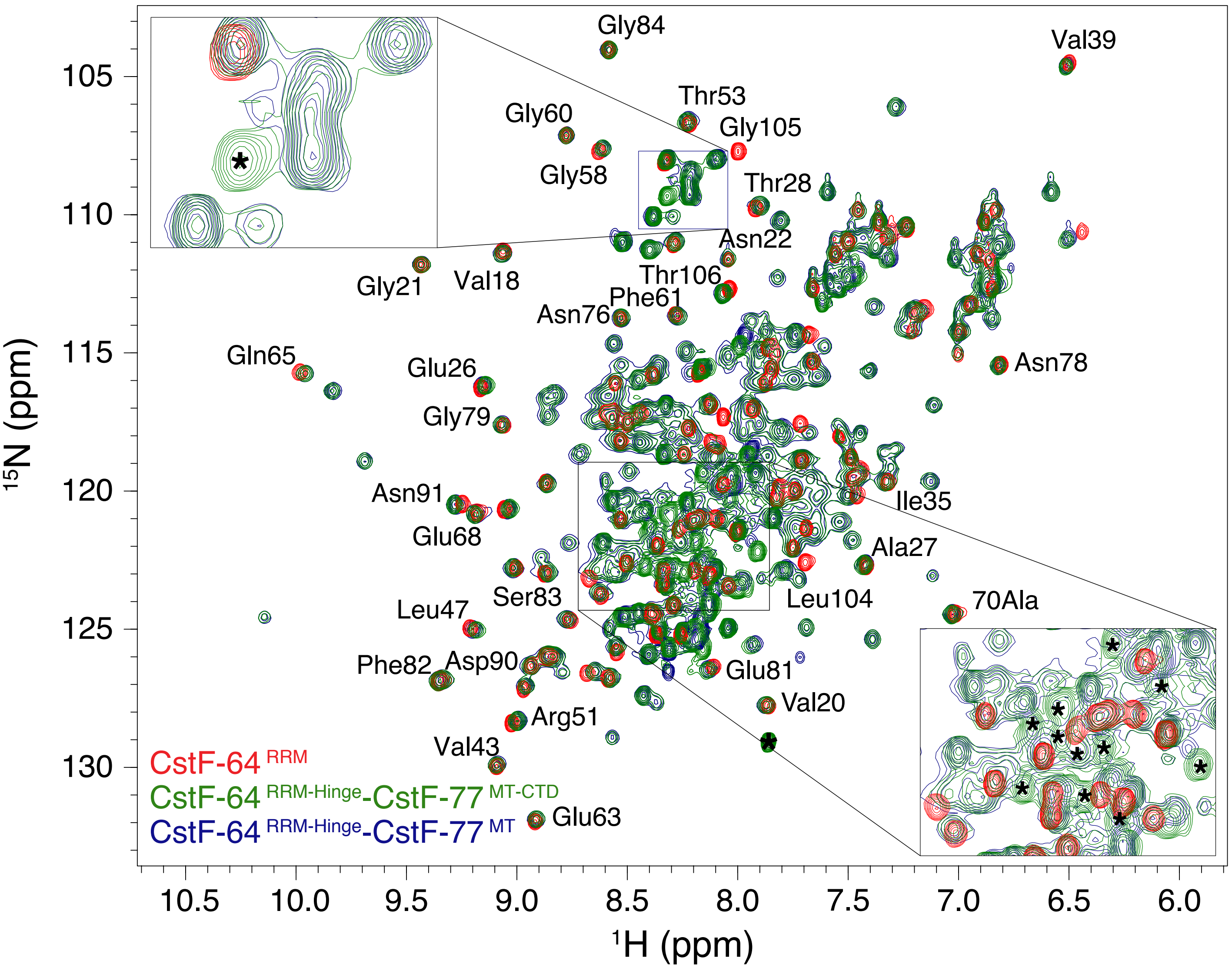

Figure S5. The CTD of CstF-77 does not affect the structure of the RRM domain of CstF-64, related to Figure 7. Overlay of 2D ^{15}N-^{1}H HSQC spectra for the isolated CstF-64^{RRM} (red contours), CstF-64$^{RRM\text{-}Hinge}$-CstF-77$^{MT\text{-}CTD}$ (green contours), and CstF-64$^{RRM\text{-}Hinge}$-CstF-77^{MT} (blue contours). CstF-64^{RRM} assignments are from Varani and co-workers (Pérez Cañadillas and Varani, *EMBO J.* **22,** 2821–2830, 2003). Peaks from the CTD of CstF-77 are denoted by asterisks. Data were collected at 600 MHz and 30°C.

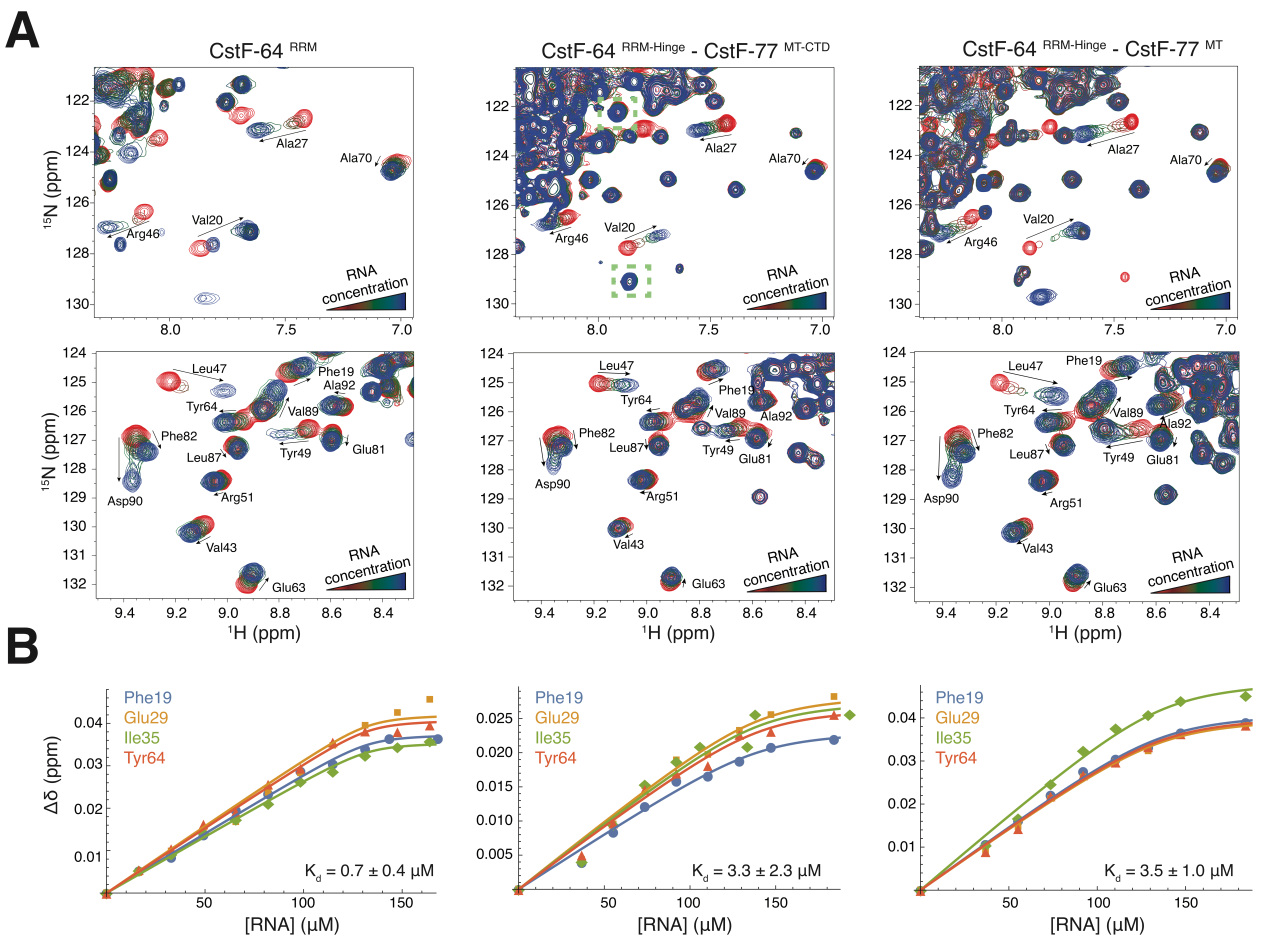

Figure S6. CstF-77 ^{CTD} affects SVL RNA binding, related to Figure 7. **(A)** Overlay of 2D ^{15}N-^{1}H HSQC spectra for titrations of CstF-64^{RRM} (*left*), CstF-64$^{RRM\text{-}Hinge}$-CstF-77$^{MT\text{-}CTD}$ (*middle*), and CstF-64$^{RRM\text{-}Hinge}$-CstF-77^{MT} (*right*). The red-green-blue contour gradient represents increasing concentrations of the SVL RNA. Two CstF-77^{CTD} peaks are highlighted by green dashed boxes in the *middle* panel. **(B)** Representative plots of the amide CSPs *vs* [RNA] used to determine K_d values at 30°C. For each construct, data are shown for Phe19 (blue circles), Glu29 (yellow squares), Ile35 (green diamonds), and Tyr64 (orange triangles).
